# The GP2a 91/97/98 amino acid substitutions play critical roles in determining PRRSV tropism and infectivity but do not affect immune responses

**DOI:** 10.1128/jvi.00048-25

**Published:** 2025-03-12

**Authors:** Ming Qiu, Shuai Li, Shubin Li, Zhe Sun, Hong Lin, Shuai Yang, Meng Cui, Yuejia Qiu, Wenhao Qi, Xiuling Yu, Shaobin Shang, Kegong Tian, François Meurens, Jianzhong Zhu, Nanhua Chen

**Affiliations:** 1 College of Veterinary Medicine, Yangzhou University 614704 , Yangzhou, China; 2 National Research Center for Veterinary Medicine 648212 , Luoyang, China; 3 Joint International Research Laboratory of Agriculture and Agri-Product Safety 614792 , Yangzhou, China; 4 Swine and Poultry Infectious Diseases Research Center, University of Montreal 70354 , Saint-Hyacinthe, Québec, Canada; 5 Jiangsu Co-Innovation Center for Prevention and Control of Important Animal Infectious Diseases and Zoonoses, Yangzhou University 38043 , Yangzhou, China; 6 Comparative Medicine Research Institute, Yangzhou University 38043 , Yangzhou, China; University of Michigan Medical School, Ann Arbor, Michigan, USA

**Keywords:** PRRSV isolates, GP2a substitutions, tropism, infectivity, immune responses

## Abstract

Porcine reproductive and respiratory syndrome virus (PRRSV) isolates share a restricted cellular tropism. Marc-145 cells derived from African green monkey are one of the few cell lines supporting PRRSV propagation *in vitro* and are commonly used for PRRS vaccine development. However, currently prevalent PRRSV isolates display different Marc-145 cell tropism while the exact determinant is not clarified yet. In this study, we identified for the first time that the 91/97/98 amino acid (aa) substitutions in GP2a of PRRSV play critical roles in determining Marc-145 adaptation. Specifically, multiple series of chimeric viruses were constructed based on four PRRSV infectious clones including Marc-145 adaptive HP-PRRSV-2 strain and Marc-145 non-adaptive NADC34-like PRRSV-2, NADC30-like PRRSV-2, and PRRSV-1 strains. The GP2a 91/97/98 aa substitutions are a sufficient and necessary determinant in NADC34-like and NADC30-like PRRSV-2, a sufficient but not necessary determinant in HP-PRRSV-2, a necessary but not sufficient determinant in PRRSV-1, respectively. In addition, the GP2a substitutions also influenced PRRSV infectivity in PAMs and piglets. Noticeably, the GP2a substitutions did not significantly affect the levels of neutralizing antibodies, porcine T follicular helper (Tfh) cells, and PRRSV-specific IFNγ secreting cells. Overall, our results not only provide new insights into PRRSV tropism and infectivity but also will facilitate PRRS vaccine development.

**IMPORTANCE:**

Prevalent PRRSV isolates present different cell tropisms *in vitro*. Clarifying the exact determinant of PRRSV tropism is crucial for PRRSV isolation and vaccine development. By constructing chimeric viruses based on four representative PRRSV infectious clones, we identified for the first time that the 91/97/98 amino acid substitutions in GP2a play critical but distinct roles in determining Marc-145 cell tropism for different PRRSV strains. The GP2a 91/97/98 amino acid substitutions also affect PRRSV infectivity in PAMs and piglets but do not influence immune responses. This study not only deciphers an exact determinant of PRRSV tropism and infectivity but also has guiding significance for PRRS vaccine development.

## INTRODUCTION

Porcine reproductive and respiratory syndrome (PRRS) is one of the most economically significant infectious diseases worldwide. The annual cost of PRRS for the global swine industry is approximately $2.7 billion (1). PRRS has jeopardized the swine industry for three decades, which causes reproductive disorders (late abortion, stillbirth, and mummified birth) in sows and respiratory distress syndromes (interstitial pneumonia and dyspnea) in all ages of pigs (2). The causative agent, PRRS virus (PRRSV), is an enveloped, single-stranded positive-sense RNA virus belonging to the genus *Betaarterivirus* in the family *Arteriviridae* (3). PRRSV genome is about 15 kb in size containing at least 10 open reading frames (ORFs). The ORF1a and ORF1b encode two polyproteins (pp1a and pp1ab) that can be processed into 16 nonstructural proteins (nsp1α, nsp1β, nsp2N, nsp2TF, nsp2 - nsp6, nsp7α, nsp7β, and nsp8–nsp12). ORFs 2–7 encode eight structural proteins including minor envelope proteins (GP2a, E, GP3, GP4, and 5a), major envelope proteins (GP5 and M), and a nucleocapsid protein (N) (2). PRRSV was first isolated in the Netherlands in 1991 and then in the United States in 1992 (4, 5). Currently, a huge amount of PRRSV strains have been isolated, which are split into two species: *Betaarterivirus suid 1* (PRRSV-1) and *Betaarterivirus suid 2* (PRRSV-2) (3). PRRSV-1 isolates can be divided into four subtypes (6). PRRSV-2 isolates have been clustered within 11 lineages (7). In China, PRRSV-1 isolates have been sporadically detected since 2006 (8). PRRSV-2 isolates are currently predominant in Chinese swine herds, mainly including highly pathogenic PRRSV-2 (HP-PRRSV-2, lineage 8) emerged in 2006 (9), NADC30-like PRRSV-2 (lineage 1.8) detected in 2013 (10), and NADC34-like PRRSV-2 (lineage 1.5) since 2017 (11).

Even though PRRSV isolates present high genetic diversity, they share a restricted host and cellular tropism. Domestic and feral pigs of *Sus scrofa* are the only known natural hosts for PRRSV. Only the differential cells of macrophage and monocyte lineages such as porcine alveolar macrophages (PAMs), monocyte-derived dendritic cells (moDCs), and pulmonary intravascular macrophages (PIMs) are permissive to PRRSV infection *in vivo* (12, 13). MA104, CL2621, and Marc-145 cells derived from African green monkeys can be utilized for PRRSV propagation *in vitro* (14, 15). PRRSV enters the target cells via receptor-mediated endocytosis, and cellular receptors are the major host determinants of PRRSV tropism (16). Several receptors/factors have been identified to play roles in PRRSV entry including heparin sulfate, sialoadhesin (CD169), CD163, CD151, DC-SIGN, vimentin, Siglec 10, and MYH9 (17–23). CD169 and CD163 are considered the main receptors for PRRSV entry in PAMs. CD169 can interact with major envelope proteins GP5 and M to mediate viral internalization (18, 24). Co-expression of CD169 and CD163 in non-permissive cells increases 10–100 times with PRRSV production compared to cells only expressing CD163, indicating that CD169 plays an important role in PRRSV entry (25). However, CD169 is not required for PRRSV infection in pigs (26). By contrast, CD163 may interact with minor envelope proteins GP2a and GP4 to facilitate viral uncoating (25, 27). A chimeric PRRSV containing ORFs 2–4 from equine arteritis virus (EAV) obtained a broad tropism in cultured cells as EAV but lost the ability to infect PAMs (16). In addition, CD163 knockout pigs are completely resistant to PRRSV infection (28). Noticeably, expression of CD163 from different host species (human, monkey, and pig) renders non-permissive cells susceptible to PRRSV infection (22). Meanwhile, CD163 also serves as a dynamic barrier to host-switching for *Arteriviruses* (29). All these results indicated that minor envelope proteins are the major viral determinants of PRRSV tropism.

Marc-145 cells are the most extensively used cells for PRRSV isolation and PRRS vaccine development (15, 30). However, an increasing number of PRRSV isolates fail to infect Marc-145 cells, including PRRSV-1 and lineage 1 PRRSV-2 (NADC30-like and NADC34-like) isolates (8, 31). The key amino acid (aa) determinant for PRRSV adaptation to Marc-145 cells remains controversial. In PRRSV-1, the 88/95 aa of GP2a is critical to improve viral growth *in vitro* (32). The 88/94/95 aa in GP2a is important for Marc-145 cell tropism (33). In PRRSV-2, GP2a-GP3 determines Marc-145 adaptation (34). The GP2a 160th and 98th aa are crucial for PRRSV-2 tropism (35, 36). Hence, clarifying the exact determinant of PRRSV tropism has guiding significance for next-generation vaccine development.

In this study, we explored the key determinants of Marc-145 cell tropism using chimeric viruses constructed based on NADC34-like PRRSV-2, NADC30-like PRRSV-2, HP-PRRSV-2, and PRRSV-1 infectious clones. Our results directly verified that the 91/97/98 aa substitutions in GP2a of PRRSV play critical roles in determining Marc-145 adaptation. In addition, the GP2a 91/97/98 aa substitutions also affected PRRSV infectivity in PAMs and piglets but did not influence immune responses. These findings not only provide new insights into PRRSV tropism and infectivity but also facilitate PRRS vaccine development.

## RESULTS

### Currently prevalent PRRSV isolates display distinct Marc-145 cell tropism

**Fig 1:**
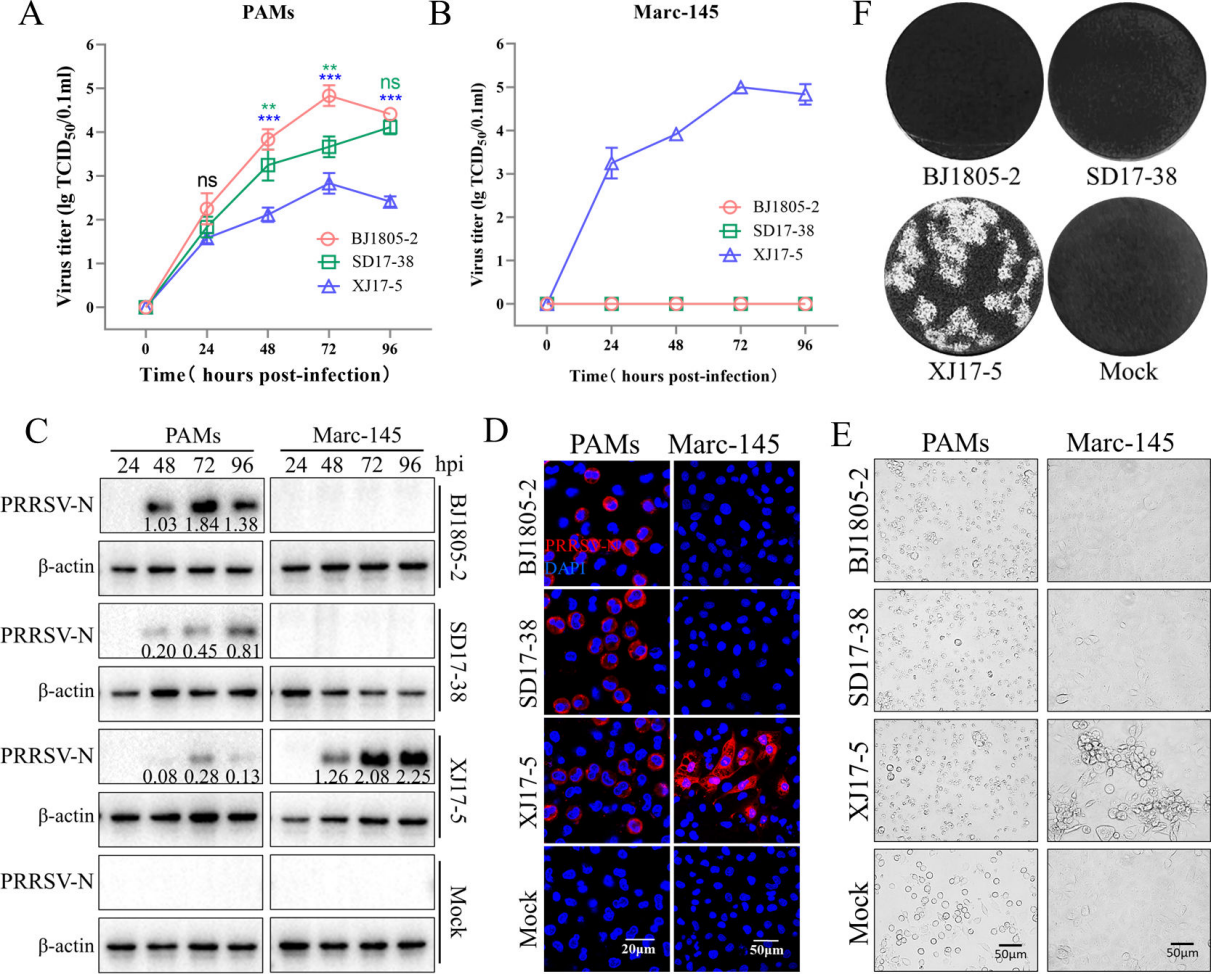
Prevalent PRRSV isolates display different cellular tropisms. (A and B) NADC34-like BJ1805-2, NADC30-like SD17-38, and HP-PRRSV-2 XJ17-5 were inoculated in PAMs and Marc-145 cells at 0.01 MOI. Supernatants were harvested at 0, 24, 48, 72, and 96 hpi for viral titration. (C) Cell samples collected at 24, 48, 72, and 96 hpi were subjected to WB detection. (D) IFA was performed at 72 hpi to detect PRRSV-N protein in infected cells. Scale bars: 20 µm (PAMs), 50 µm (Marc-145). (E) CPE was recorded at 72 hpi. Scale bars: 50 µm. (F) Plaque assay was used to detect Marc-145 adaptation for serially diluted PRRSV isolates. One representative plaque for each virus was shown.

At first, we determined the *in vitro* cellular tropism of currently prevalent PRRSV-2 isolates (NADC34-like BJ1805-2 strain, NADC30-like SD17-38 strain, and HP-PRRSV-2 XJ17-5 strain). PAMs were permissive to all these isolates but yielded distinct viral titers (Fig. 1A). NADC34-like BJ1805-2 had the highest replication efficacy in PAMs followed by NADC30-like SD17-38 and HP-PRRSV-2 XJ17-5, respectively. Remarkably, Marc-145 cells were only susceptible to the XJ17-5 isolate but not to the SD17-38 and BJ1805-2 strains (Fig. 1B). Similarly, PRRSV N protein could be detected in BJ1805-2-, SD17-38-, and XJ17-5-infected PAMs but only in XJ17-5-infected Marc-145 cells during WB and IFA detections (Fig. 1C and D). In addition, cytopathic effects (CPE) and plaques could only be observed in XJ17-5 infected but not in SD17-38- and BJ1805-2-infected Marc-145 cells at 72 hpi (Fig. 1E and F). Moreover, three sources of Marc-145 cells were tested, and the same results were obtained (data not shown). These results confirmed that the HP-PRRSV-2 XJ17-5 isolate is Marc-145 adaptive while NADC34-like BJ1805-2 and NADC30-like SD17-38 strains are Marc-145 non-adaptive.

### The 91/97/98 aa substitutions in GP2a are a sufficient and necessary determinant for NADC34-like and NADC30-like PRRSV-2 adaptation to Marc-145 cells

**Fig 2:**
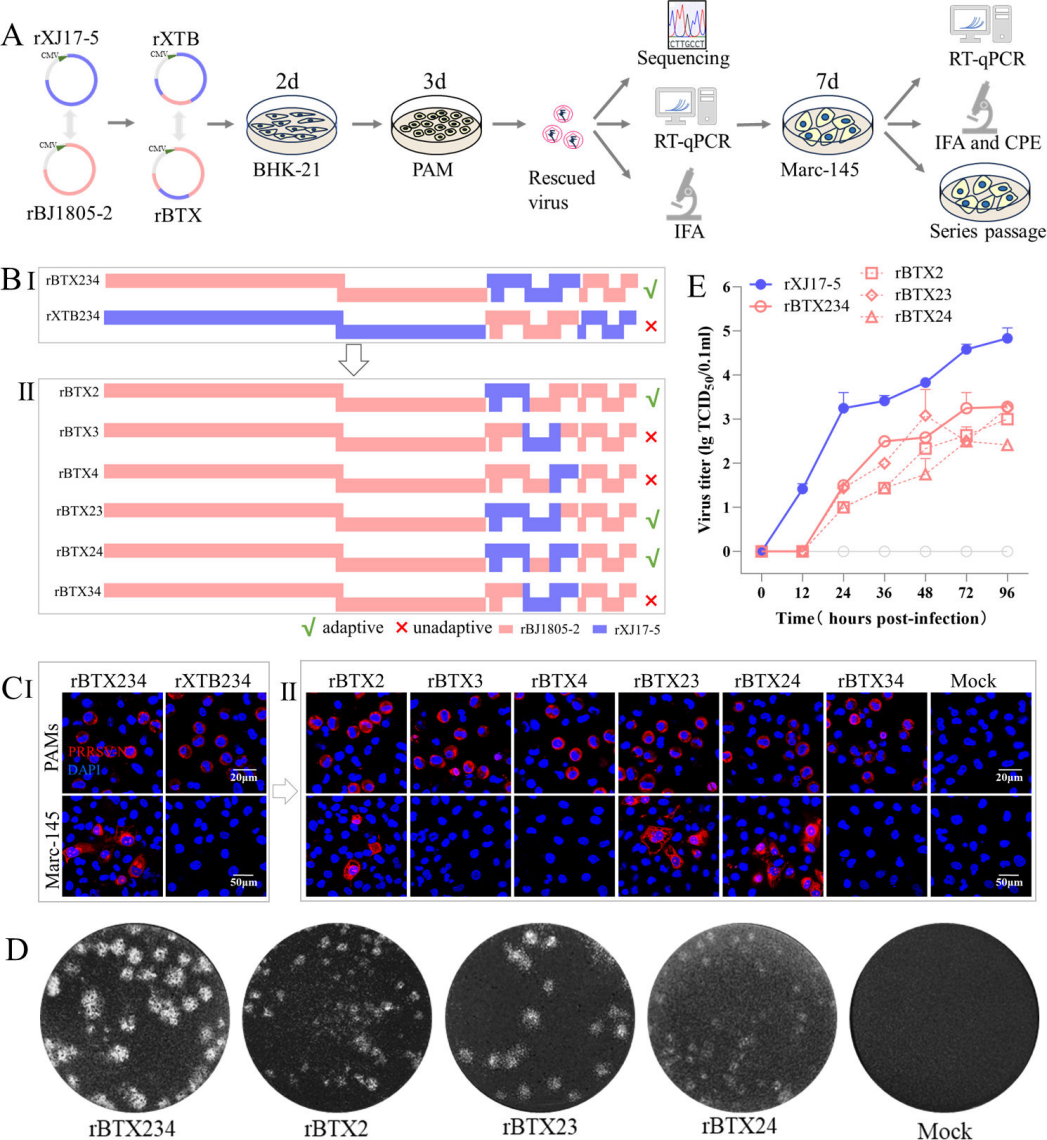
GP2a is a critical determinant in NADC34-like rBJ1805-2 strain for Marc-145 cell tropism. (A) Experimental flow: construct PRRSV infectious clones (rBJ1805-2 and rXJ17-5), generate chimeric clones (rXTB and rBTX), transfect into BHK-21 cells, rescue chimeric viruses in PAMs, and then determine the Marc-145 cell tropism. (B) Strategies to construct chimeric viruses switching minor envelope protein-encoding genes between rBJ1805-2 and rXJ17-5. (C) IFA detection of infected PAMs and Marc-145 cells at 3 dpi and 7 dpi, respectively. Scale bars: 20 µm (PAMs), 50 µm (Marc-145). (D) Serially diluted chimeric viruses were used to identify Marc-145 adaptation via plaque assay. A representative plaque for each Marc-145 adaptive virus was shown. (E) Marc-145 cell adaptive viruses were inoculated into Marc-145 cells at 0.01 MOI. The supernatants were collected at 0, 12, 24, 36, 48, 72, and 96 hpi for virus titration. rXJ17-5 was set as a positive control.

To explore the key determinant of PRRSV tropism to Marc-145 cells, we started with the constructions of NADC34-like PRRSV-2 BJ1805-2 and HP-PRRSV-2 XJ17-5 infectious clones (rBJ1805-2 and rXJ17-5) that maintain the same Marc-145 cell tropism as parental viruses (Fig. S1). Previous studies have shown that minor envelope proteins encoded by ORFs 2–4 genes are the major determinant of PRRSV tropism (16, 27). Therefore, we first constructed chimeric viruses switching ORFs 2–4 genes of Marc-145-adapted rXJ17-5 and Marc-145-non-adapted rBJ1805-2 infectious clones (Fig. 2A and B-I). IFA results showed that the chimeric rBJ1805-2 virus containing ORFs 2–4 from rXJ17-5 (named rBTX234) obtained Marc-145 adaptation. By contrast, the chimeric rXJ17-5 virus containing ORFs 2–4 from rBJ1805-2 (named rXTB234) lost Marc-145 adaptation (Fig. 2C–I). These results confirmed that minor envelope proteins are the determinant of Marc-145 cell tropism for NADC34-like rBJ1805-2 strain.

Subsequently, we constructed six chimeric rBJ1805-2 viruses containing one or two minor envelope protein-encoding genes (ORF2, ORF3, and ORF4) from rXJ17-5, respectively (Fig. 2B-II). Three chimeric viruses (rBTX2, rBTX23, and rBTX24) containing the ORF2 gene from rXJ17-5 obtained Marc-145 adaptation, while the other three chimeric viruses (rBTX3, rBTX4, and rBTX34) that did not have the rXJ17-5 ORF2 gene could not infect Marc-145 cells (Fig. 2C-II). Results from growth curves and plaque assays further supported this phenomenon (Fig. 2D and E), which indicated that GP2a encoded by the ORF2 gene is the key determinant of Marc-145 adaptation for the rBJ1805-2 virus.

**Fig 3:**
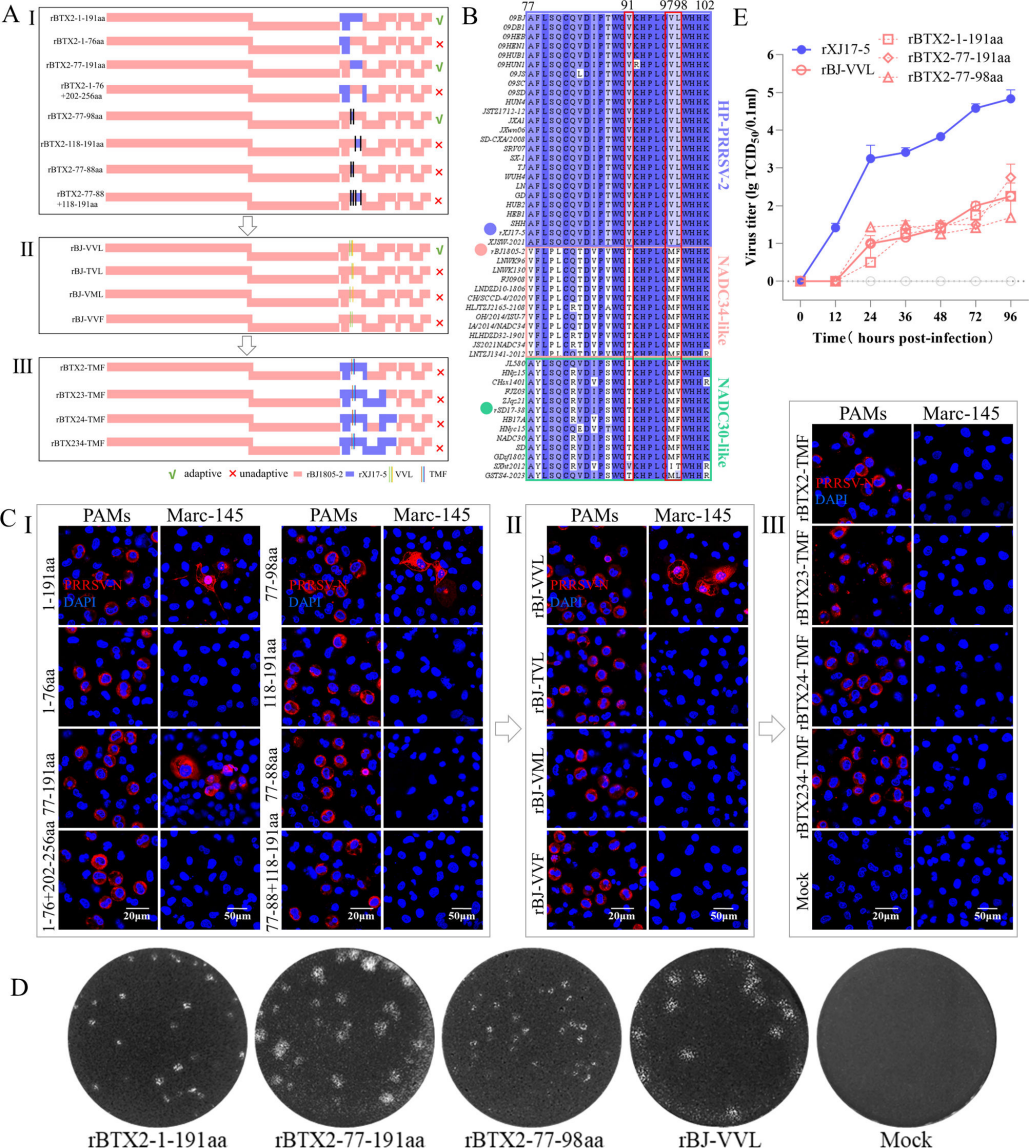
GP2a 91/97/98 aa substitutions determine Marc-145 adaptation for rBJ1805-2 strain. (A) The strategies for fragment replacements and point mutations within GP2a of rBJ1805-2 strain. (A-I) fragment replacement. (A-II) The GP2a 91/97/98 aa substitutions in rBJ1805-2 were mutated from "TMF" to "VVL," and then single reverse point mutations containing "TVL," "VML," and "VVF" patterns were also constructed. (A-III) The GP2a 91/97/98 aa substitutions in Marc-145-adapted rBTX2, rBTX23, and rBTX24 strains were mutated to the "TMF" pattern. (B) The amino acid sequence alignment of 77–102 aa in GP2a of HP-PRRSV-2, NADC34-like, and NADC30-like PRRSV-2 strains. The blue, red, and green dots indicate the rXJ17-5, rBJ1805-2, and rSD17-38 strains, respectively. (C) IFA detection of infected PAMs and Marc-145 cells at 3 dpi and 7 dpi, respectively. Scale bars: 20 µm (PAMs), 50 µm (Marc-145). (D) Serially diluted chimeric viruses were detected by plaque assay for Marc-145 adaptation. A representative plaque for each Marc-145 adaptive virus was shown. (E) Marc-145 cell adaptive viruses (0.01 MOI) were submitted to virus titration using supernatants collected at 0, 12, 24, 36, 48, 72, and 96 hpi. rXJ17-5 was set as a positive control.

Moreover, chimeric viruses containing fragment replacements were constructed (Fig. 3A-I). Chimeric rBJ1805-2 viruses (rBTX2-1-191aa, rBTX2-77-191aa, and rBTX2-77-98aa) containing rXJ17-5 sequences encoding GP2a 1–191 aa, 77–191 aa, and 77–98 aa obtained Marc-145 cell tropism. However, the chimeric virus (rBTX2-77-88aa) containing rXJ17-5 sequences encoding GP2a 77–88 aa did not obtain Marc-145 adaptation (Fig. 3C-I), suggesting that GP2a 89–98 aa is the determinant region. Sequence comparison showed that there are only three conserved aa substitutions (91/97/98) in this region (Fig. 3B). Noticeably, the GP2a 91/97/98 aa patterns were “VVL” in Marc-145-adapted HP-PRRSV-2 strains (containing rXJ17-5) and were mainly “T/IMF” in Marc-145-non-adapted NADC34-like and NADC30-like PRRSV-2 strains (containing rBJ1805-2). Therefore, the mutated rBJ1805-2 virus (rBJ-VVL) switching the “TMF” pattern to the “VVL” pattern was constructed, which gained Marc-145 tropism, indicating that the GP2a 91/97/98 “VVL” pattern is a sufficient determinant. In addition, the mutated viruses (rBJ-TVL, rBJ-VML, and rBJ-VVF) containing either two of the three substitutions could not infect Marc-145 cells (Fig. 3A-II and C-II), suggesting that the GP2a 91/97/98 aa substitutions are the minimum determinant. Furthermore, the GP2a 91/97/98 “VVL” in Marc-145 adaptive chimeric rBJ1805-2 viruses containing ORF2, ORF2 +3, ORF2 +4, ORF2 +3 + 4 from rXJ17-5 were mutated back to “TMF,” and all the mutated chimeric viruses (rBTX2-TMF, rBTX23-TMF, rBTX24-TMF, and rBTX234-TMF) lost Marc-145 adaptation again (Fig. 3A-III and B-III). The results from plaque assays and growth curves also supported that GP2a 91/97/98 aa substitutions are the sufficient and necessary determinant of Marc-145 adaptation in NADC34-like PRRSV-2 rBJ1805-2 strain (Fig. 3D and E).

**Fig 4:**
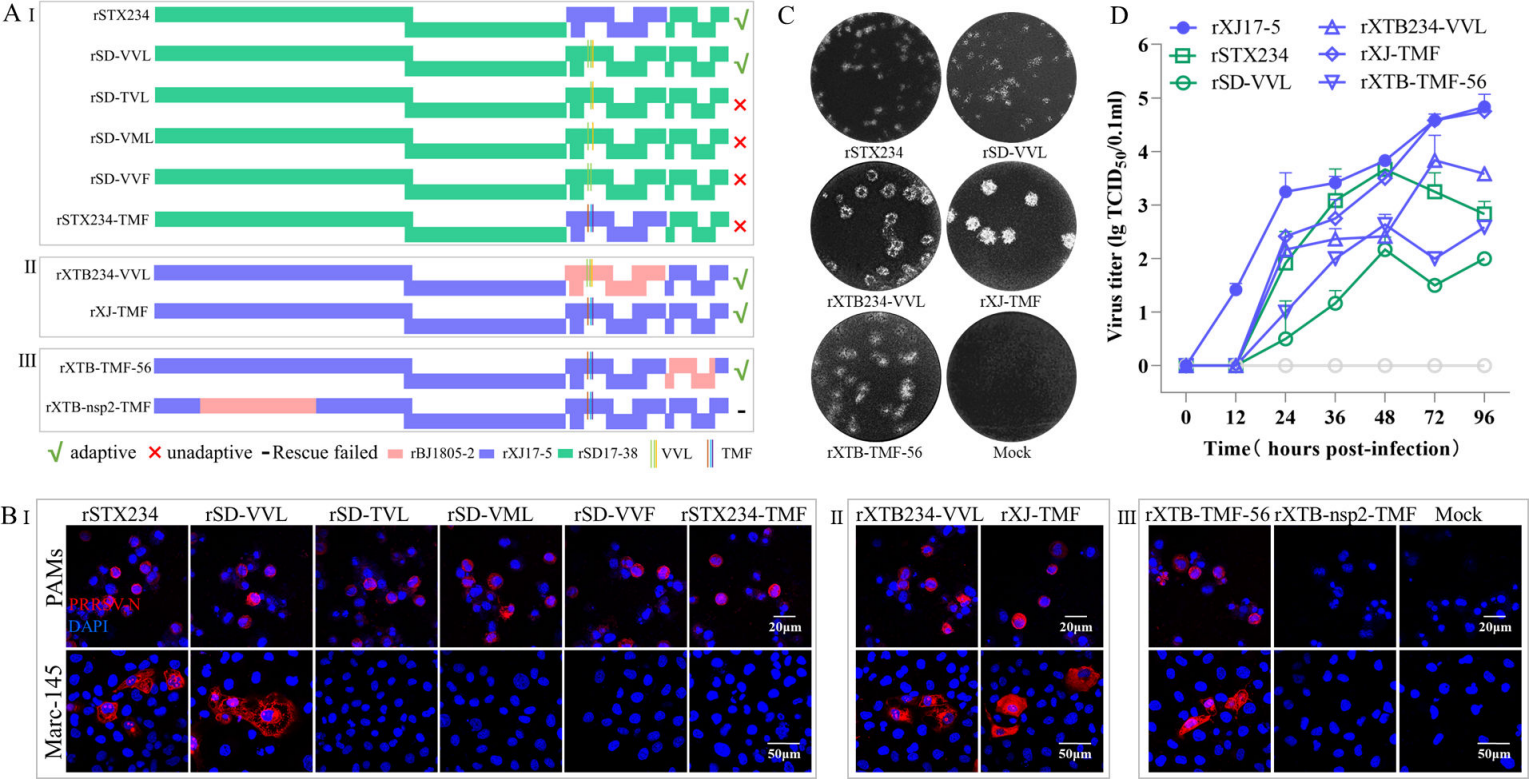
Influence of GP2a 91/97/98 aa substitutions on Marc-145 adaptation for NADC30-like rSD17-38 and HP-PRRSV-2 rXJ17-5 strains. (A) Construction strategies. (A-I) The minor envelope proteins and GP2a 91/97/98 aa in rSD17-38 were replaced with corresponding regions in rXJ17-5, respectively. Single reverse point mutations containing "TVL," "VML," and "VVF" patterns were also constructed, respectively. In addition, the GP2a 91/97/98 “VVL” pattern in rSTX234 was also mutated back to the “TMF” pattern as in rSD17-38. (A-II) The GP2a 91/97/98 “TMF” pattern in rXTB234 was mutated to the “VVL” pattern, and the GP2a 91/97/98 “VVL” pattern in rXJ17-5 was mutated to the “TMF” pattern. (A-III) The major envelope proteins GP5-M in rXJ-TMF were replaced with corresponding regions from rBJ1805-2. The nsp2 in rXJ-TMF was replaced with nsp2 from rBJ1805-2. (B) IFA detection of infected PAMs and Marc-145 cells at 3 dpi and 7 dpi, respectively. Scale bars: 20 µm (PAMs), 50 µm (Marc-145). (C) Plaque assay detection of serially diluted viruses. A representative plaque for each Marc-145 adaptive virus was shown. (D) Marc-145 cell adaptive viruses (0.01 MOI) were submitted to virus titration using supernatants collected at 0, 12, 24, 36, 48, 72, and 96 hpi. rXJ17-5 was set as a positive control.

Both NADC34-like PRRSV-2 and NADC30-like PRRSV-2 belong to lineage 1 PRRSV-2. Therefore, we further evaluated the influence of the GP2a 91/97/98 aa pattern on Marc-145 adaptation in the NADC30-like rSD17-38 infectious clone (Fig. S1). The results showed that both the chimeric rSD17-38 virus containing ORFs 2–4 from rXJ17-5 (rSTX234) and the mutated rSD17-38 virus only containing GP2a 91/97/98 “VVL” substitutions (rSD-VVL) obtained Marc-145 adaptation (Fig. 4A-I). Moreover, the mutated viruses (rSD-TVL, rSD-VML, and rSD-VVF) containing either two of the three substitutions could not infect Marc-145 cells (Fig. 4A-I). Meanwhile, the mutated rSTX234 strain with GP2a 91/97/98 aa reversing back to “TMF” (rSTX234-TMF) lost Marc-145 tropism again (Fig. 4A-I). These results indicated that the GP2a 91/97/98 aa substitutions are also a sufficient and necessary determinant of Marc-145 tropism in NADC30-like rSD17-38 strain (Fig. 4). All the above results supported that 91/97/98 aa substitutions in GP2a are a sufficient and necessary determinant of Marc-145 adaptation in lineage 1 PRRSV-2 isolates.

### The GP2a 91/97/98 aa substitutions are a sufficient but not necessary determinant for HP-PRRSV-2 adaptation to Marc-145 cells

Even though the chimeric HP-PRRSV-2 rXJ17-5 (rXTB234) containing ORFs 2–4 from rBJ1805-2 lost Marc-145 adaptation (Fig. 2B and C), when the GP2a 91/97/98 aa substitutions were reversed back to “VVL,” the mutated chimeric virus (rXTB234-VVL) obtained Marc-145 adaptation again (Fig. 4A-II). The results supported that the GP2a 91/97/98 aa substitutions are also a sufficient determinant of Marc-145 adaptation for HP-PRRSV-2 rXJ17-5 strain. Noticeably, the mutated rXJ17-5 strain only containing the “TMF” pattern (rXJ-TMF) still maintained Marc-145 cell tropism (Fig. 4A-II), indicating that the GP2a 91/97/98 aa substitutions are not a necessary determinant of Marc-145 adaptation in HP-PRRSV-2 (Fig. 4A through D).

Besides the minor envelope proteins, major envelope proteins (GP5 and M) and nsp2 have also been reported to play roles in PRRSV tropism (24, 37, 38). Therefore, we evaluated the influence of major envelope proteins (GP5 and M) and nsp2 on HP-PRRSV-2 Marc-145 cell tropism. The chimeric virus (rXTB-TMF-56) containing GP5 and M encoding genes from Marc-145 non-adapted strain (rBJ1805-2) could still infect Marc-145 cells (Fig. 4A-III), suggesting that major envelope proteins are not critical for Marc-145 adaptation (Fig. 4A through D). The chimeric virus (rXTB-TMF-nsp2) containing the nsp2 encoding gene from Marc-145 non-adapted strain (rBJ1805-2) could not be rescued (Fig. 4A and B). Therefore, we continued to explore the additional sufficient determinants for HP-PRRSV-2 within the minor envelope proteins.

**Fig 5:**
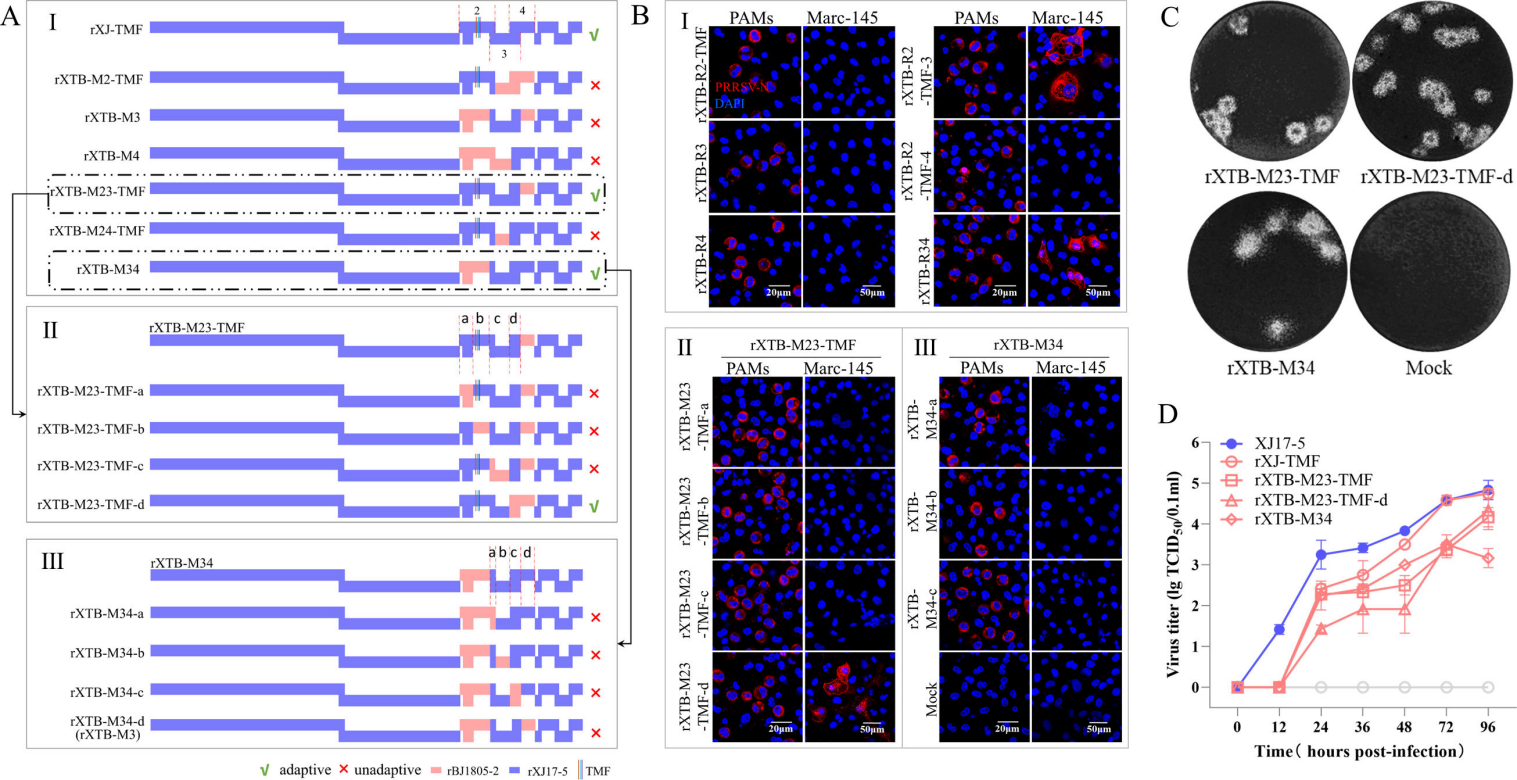
Identification of additional sufficient determinants of Marc-145 cell tropism in minor envelope proteins of HP-PRRSV-2 rXJ17-5 strain. (A) Construction strategies. (A-I) Each minor envelope protein in rXJ-TMF was mutated individually or combined. (A-II) The GP2a-GP3 region in rXTB-M23-TMF was divided into four fragments a, b, c, and d that were individually replaced with rBJ1805-2 counterpart. (A-III) The GP3-GP4 region in rXTB-M34 was divided into four fragments a, b, c, and d that were individually replaced with the corresponding regions in rBJ1805-2. (B) IFA detection of infected PAMs and Marc-145 cells at 3 dpi and 7 dpi, respectively. Scale bars: 20 µm (PAMs), 50 µm (Marc-145). (C) Plaque assay detection of serially diluted viruses. A representative plaque for each Marc-145 adaptive virus was shown. (D) Marc-145 cell adaptive viruses (0.01 MOI) were submitted to virus titration using supernatants collected at 0, 12, 24, 36, 48, 72, and 96 hpi. rXJ17-5 was set as a positive control.

Based on the mutated rXJ17-5 strain (rXJ-TMF) containing GP2a 91/97/98 “TMF” pattern, we constructed three chimeric viruses (rXTB-M2-TMF, rXTB-M3, and rXTB-M4) to evaluate which one of the minor envelope proteins (GP2a, GP3, or GP4) contains the other sufficient determinants for Marc-145 adaptation. However, all of them lost Marc-145 cell tropism (Fig. 5A-I and B-I). Subsequently, we constructed chimeric viruses (rXTB-M23-TMF, rXTB-M24-TMF, and rXTB-M34) maintaining two of the three minor envelope proteins. The rXTB-M23-TMF and rXTB-M34 chimeric viruses obtained Marc-145 adaptation, suggesting that GP2a-GP3 (besides the GP2a 91/97/98 aa substitutions) and GP3-GP4 regions contain the additional sufficient determinants for Marc-145 adaptation in HP-PRRSV-2 (Fig. 5A-I and B-I).

To narrow down the additional sufficient determinants, the GP2a-GP3 region in rXTB-M23-TMF virus and the GP3-GP4 region in rXTB-M34 virus were further divided into four fragments (a, b, c, and d), respectively. Serial chimeric viruses replacing each of the four fragments were constructed (Fig. 5A-II and-III). Only the rXTB-M23-TMF-d chimeric virus excluding the GP3-GP4 overlap region could still adapt to Marc-145 cells (Fig. 5A-II and B-II through D). All chimeric viruses replacing either fragment in the rXTB-M34 backbone lost Mar-145 cell tropism (Fig. 5A-III and B-III). Herein, the GP2a 91/97/98 aa substitutions, GP2a-GP3 (without GP2a 91/97/98 aa substitutions and GP3-GP4 overlap region), and GP3-GP4 in HP-PRRSV-2 are sufficient determinants for Marc-145 adaptation.

### The GP2a 91/97/98 aa substitutions are a necessary but not sufficient determinant for PRRSV-1 adaptation to Marc-145 cells

**Fig 6:**
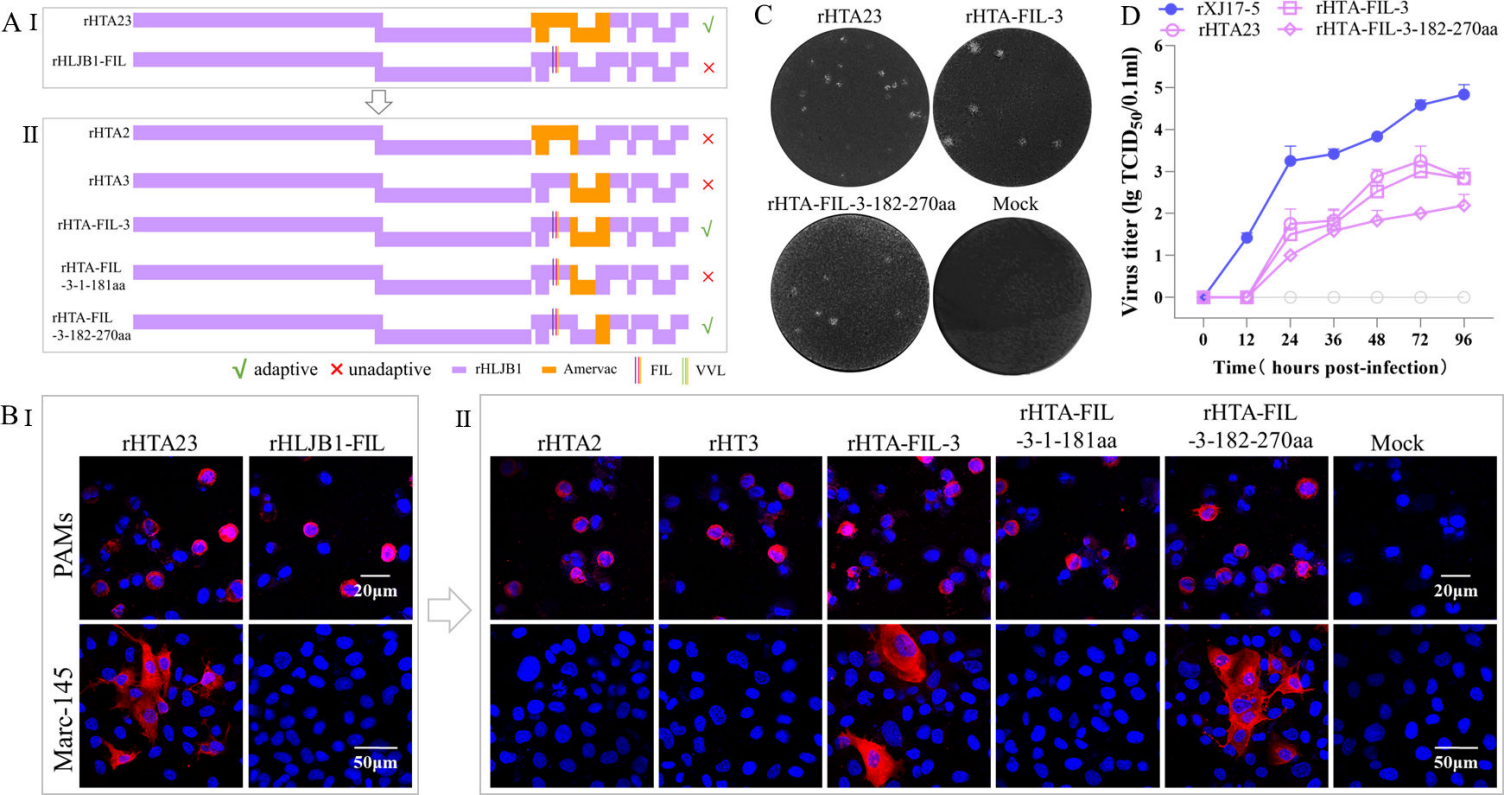
The influence of GP2a substitutions in the PRRSV-1 rHLJB1 strain on Marc-145 adaptation. (A) Fragment replacement and point mutation strategies. (B) IFA detection of infected PAMs and Marc-145 cells at 3 dpi and 7 dpi, respectively. Scale bars: 20 µm (PAMs), 50 µm (Marc-145). (C) Plaque assay detection of serially diluted viruses. A representative plaque for each Marc-145 adaptive virus was shown. (D) Marc-145 cell adaptive viruses (0.01 MOI) were submitted to virus titration using supernatants collected at 0, 12, 24, 36, 48, 72, and 96 hpi. rXJ17-5 was set as a positive control.

The corresponding positions of PRRSV-2 GP2a 91/97/98 aa in PRRSV-1 are GP2a 88/94/95 aa, which have shown to be responsible for Marc-145 adaptation in PRRSV-1 13V091 and IVI-1173 isolates (33). However, both 13V091 and IVI-1173 strains are already adaptive to Marc-145 cells. To verify whether these aa substitutions are a sufficient determinant for switching Marc-145 adaptation, this study assessed the influence of these aa substitutions in a Marc-145 non-adaptive PRRSV-1 strain (rHLJB1) (Fig. S1). Considering that besides the ORF3-ORF4 overlap region, the ORF4 gene between Marc-145 non-adaptive rHLJB1 and Marc-145 adaptive Amervac vaccine strains are exactly identical. Therefore, we only replaced ORF2-ORF3 genes (including the ORF3-ORF4 overlap region) in the rHLJB1 strain with corresponding genes from the Amervac strain (rHTA23). As shown in Fig. 6, the rHTA23 obtained Marc-145 cell tropism. When we mutated the GP2a “IMF” pattern (in rHLJB1) to the “FIL” pattern (in Amervac strain) to generate rHLJB1-FIL, the rHLJB1-FIL did not gain Marc-145 adaptation (Fig. 6A-I and B-I), indicating that the triple substitutions in GP2a of PRRSV-1 are not a sufficient determinant of Marc-145 adaptation.

We constructed two more chimeric rHLJB1 viruses containing ORF2 and ORF3 from the Amervac strain (rHTA2 and rHTA3), respectively. However, both of them could not adapt to Marc-145 cells (Fig. 6A-II and B-II). Noticeably, the chimeric virus (rHTA-FIL-3) containing GP2a “FIL” pattern and GP3 from Amervac strain obtained Marc-145 tropism (Fig. 6A through D). Therefore, we continued to construct two chimeric viruses (rHTA-FIL-3-1-181aa and rHTA-FIL-3–182-270aa) containing GP2a “FIL” pattern together with GP3 1–181aa fragment or GP3-GP4 overlap region (182–270aa) from Amervac strain (Fig. 6A-II). Our results showed that the rHTA-FIL-3–182-270aa virus obtained Marc-145 adaptation while the rHTA-FIL-3-1-181aa did not (Fig. 6B-II through D), indicating that the GP2a triple substitutions combined with GP3-GP4 overlap region are the sufficient determinant of Marc-145 cell tropism in PRRSV-1.

### The effects of GP2a 91/97/98 aa substitutions switching between PRRSV-1 and PRRSV-2 on Marc-145 adaptation

**Fig 7:**
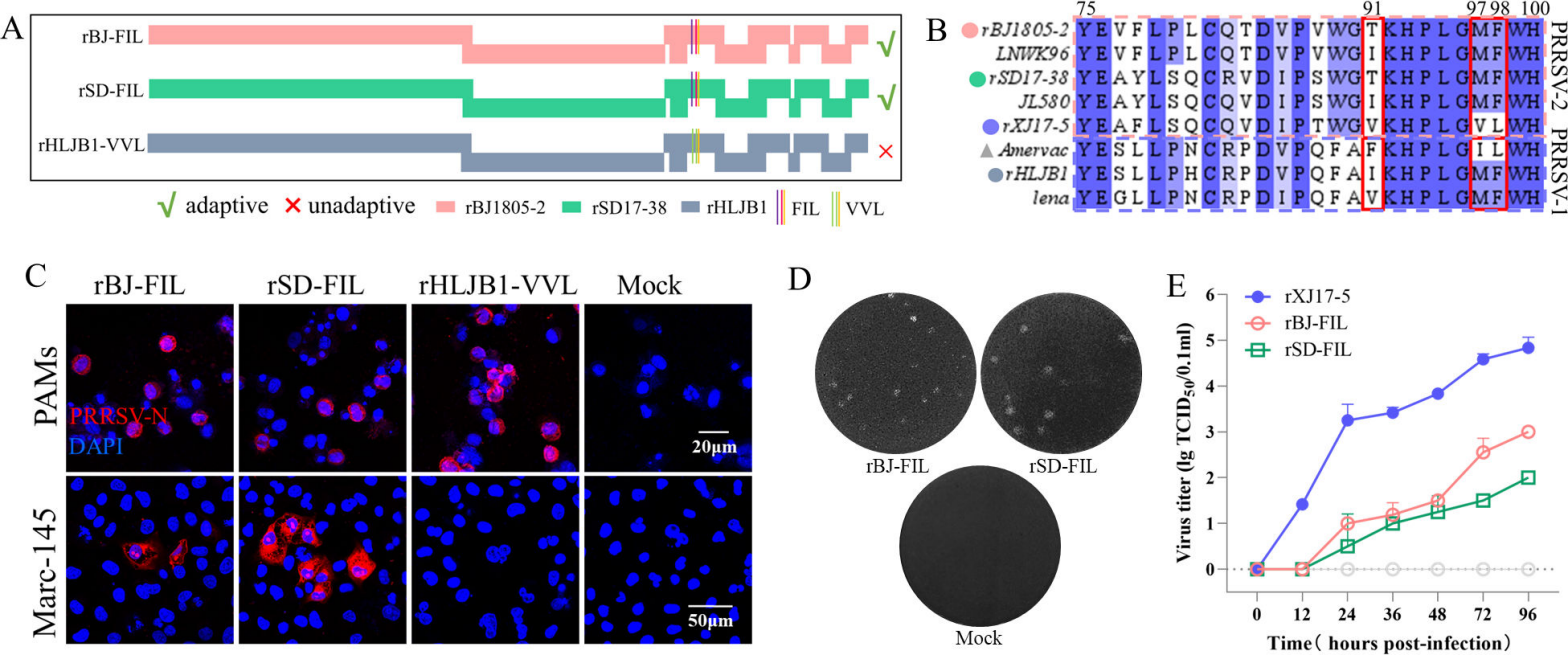
Switching GP2a 91/97/98 aa substitutions between PRRSV-1 and PRRSV-2 strains. (A) The GP2a 91/97/98 aa in PRRSV-2 strains (rBJ1805-2 and rSD17-38) were mutated to “FIL” from PRRSV-1. Meanwhile, the GP2a 91/97/98 aa in PRRSV-1 rHLJB1 strain was mutated to “VVL” pattern from PRRSV-2. (B) Amino acid sequence alignment of 75–100 aa in GP2a from PRRSV-1 and PRRSV-2 strains. The red, green, blue, and gray dots remark the rBJ1805-2, rSD17-38, rXJ17-5, and rHLJB1 strains, respectively. The “FIL” pattern is from the Marc-145-adapted PRRSV-1 Amervac strain (gray triangle). (C) IFA detection of infected PAMs and Marc-145 cells at 3 dpi and 7 dpi, respectively. Scale bars: 20 µm (PAMs), 50 µm (Marc-145). (D) Plaque assay detection of serially diluted viruses. A representative plaque for each Marc-145 adaptive virus was shown. (E) Marc-145 cell adaptive viruses (0.01 MOI) were submitted to virus titration using supernatants collected at 0, 12, 24, 36, 48, 72, and 96 hpi. rXJ17-5 was set as positive control.

Based on two Marc-145 non-adapted PRRSV-2 infectious clones (rBJ1805-2 and rSD17-38), two mutated viruses (rBJ-FIL and rSD-FIL) containing GP2a “FIL” pattern from Marc-145 adapted PRRSV-1 strain (Amervac) were constructed (Fig. 7A and B). Both mutated viruses also obtained Marc-145 cell tropism, indicating that GP2a triple substitutions from the Marc-145 adaptive PRRSV-1 strain are also sufficient to switch Marc-145 cell tropism for lineage 1 PRRSV-2 isolates (Fig. 7C through E). By contrast, another mutated PRRSV-1 strain (rHLJB1-VVL) containing GP2a 91/97/98 “VVL” pattern from Marc-145-adapted PRRSV-2 strain (rXJ17-5) was constructed (Fig. 7A and B). The rHLJB1-VVL virus was still not permissive to Marc-145 cells (Fig. 7C). Due to the inconsistent sizes of GP3-GP4 overlap regions (268 bp in PRRSV-1 and 220 bp in PRRSV-2), we did not further construct the chimeric PRRSV-1 virus containing GP2a “VVL” substitutions and GP3-GP4 overlap region from PRRSV-2. The obtained results are consistent with the above findings that the GP2a triple substitutions are a sufficient determinant of Marc-145 adaptation for lineage 1 PRRSV-2 isolates, but they are not a sufficient determinant for PRRSV-1 strain.

### The GP2a 91/97/98 aa patterns are highly diverse among PRRSV isolates

Considering the critical effects of GP2a 91/97/98 aa patterns on PRRSV tropism, we evaluated the genetic diversity of GP2a 91/97/98 aa by bioinformatics analysis using 173 and 661 GP2a sequences from PRRSV-1 and PRRSV-2 strains, respectively (Fig. S2). The GP2a 91/97/98 aa patterns are highly diverse in both PRRSV-1 and PRRSV-2. The “FMF,” “VMF,” and “IMF” are three major patterns in PRRSV-1 (Fig. S2A), while “VVL,” “TMF,” and “TML” are three major patterns in PRRSV-2 (Fig. S2B), respectively. Furthermore, previous studies have confirmed that PRRSV-1 GZ11-G1 and Amervac strains with GP2a “FIL” pattern are Marc-145 adaptive, while HLJB-1 and BJEU06-1 isolates with GP2a “I/TMF” pattern are Marc-145 non-adaptive (Fig. S2C) (8, 39, 40). Similarly, NADC30-like recombinants (FJ1402 and SCABTC-202305) and HP-PRRSV-2 isolates (XJ17-5, JXA1, and JXwn06) with GP2a “VVL” pattern are Marc-145 adaptive (Fig. S2C) (9, 41–44). Meanwhile, lineage 1 PRRSV-2 isolates (BJ1805-2, JS2021NADC34, SCcd2020, SD17-38) with GP2a “TMF” pattern are Marc-145 non-adaptive (31, 45, 46). These retrospective analysis results are exactly consistent with the chimeric virus results in this study.

### The GP2a 91/97/98 aa substitutions also affect PRRSV infectivity to PAMs

**Fig 8:**
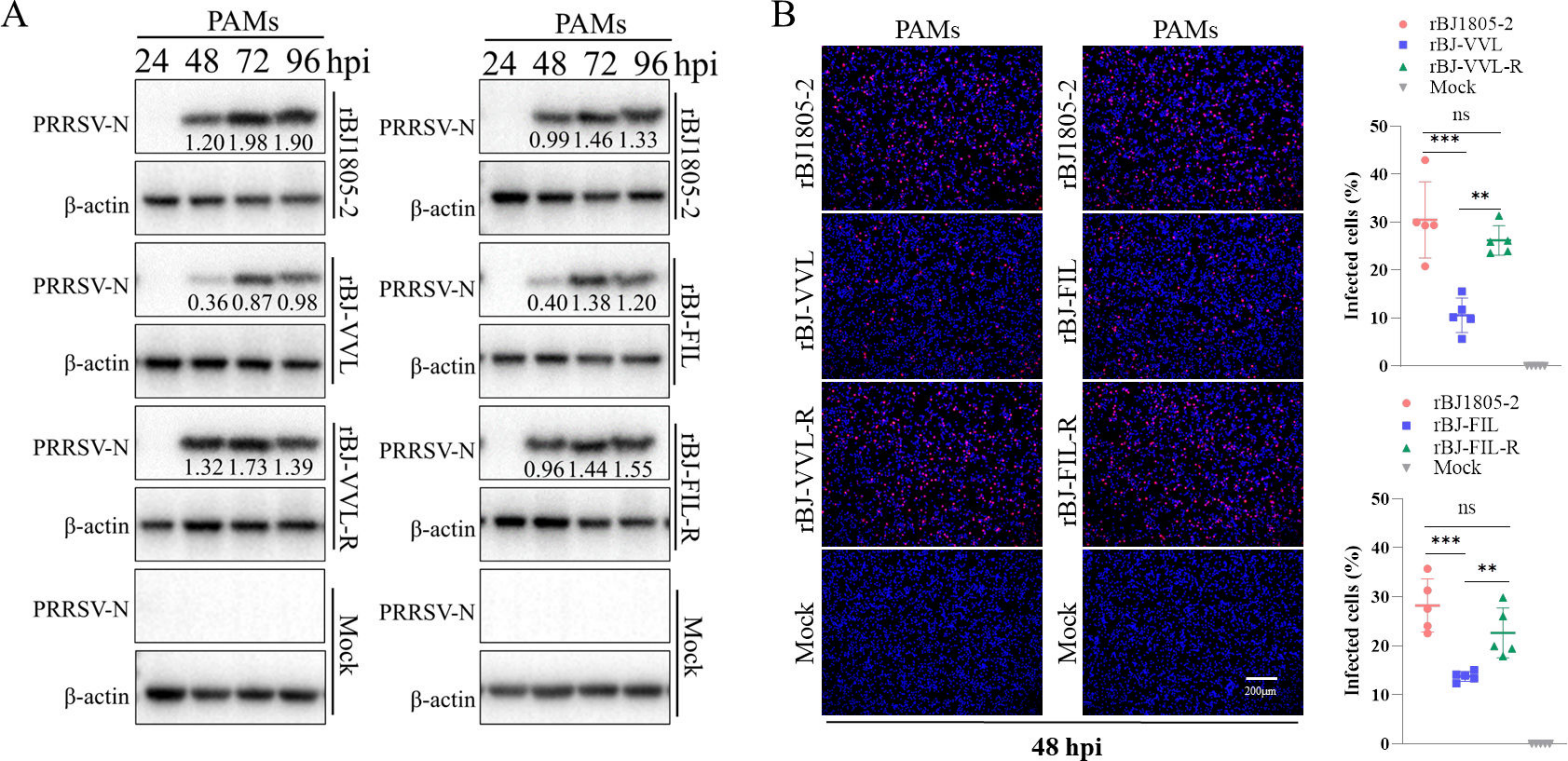
The influence of GP2a 91/97/98 aa substitutions on NADC34-like PRRSV-2 infectivity in PAMs. (A) WB results from rBJ1805-2, rBJ-VVL, and rBJ-VVL-R-infected PAMs (Left). WB results from rBJ1805-2, rBJ-FIL, and rBJ-FIL-R inoculated PAMs (Right). Cell samples were collected at 24, 48, 72, and 96 hpi for WB detection. (B) IFA detection of infected PAMs at 48 hpi. A representative image from each group was shown. The percentages of infected cells from different groups were calculated from five replicates. The viral titer used for infection is 0.01 MOI.

The Marc-145-non-adapted BJ1805-2 isolate has significantly higher replication efficacy than the Marc-145-adapted XJ17-5 isolate in PAMs (Fig. 1). To analyze whether the GP2a 91/97/98 aa substitutions play a role in PRRSV infectivity in PAMs, we compared the replication efficacies between the parental rBJ1805-2 and two mutated viruses (rBJ-VVL and rBJ-FIL) in PAMs. WB results showed that higher amounts of N proteins could be detected in rBJ1805-2 (GP2a “TMF” pattern) infected PAMs than in rBJ-VVL (GP2a “VVL” pattern) and rBJ-FIL (GP2a “FIL” pattern) infected PAMs (Fig. 8A). To confirm the decreased PRRSV replication in PAMs was induced by GP2a 91/97/98 aa substitutions, the GP2a 91/97/98 reverse mutants (rBJ-VVL-R and rBJ-FIL-R, both with GP2a “TMF” pattern) were further constructed, which showed that N protein expressions in reverse mutants obviously increased and were similar to the parental rBJ1805-2 virus (Fig. 8A). Furthermore, IFA detection of infected PAMs at 48 hpi showed that the mutated viruses (rBJ-VVL and rBJ-FIL) have significantly lower infection efficiencies than the parental virus (rBJ1805-2) and reverse mutants (rBJ-VVL-R and rBJ-FIL-R) (Fig. 8B). These results validated that the GP2a 91/97/98 aa substitutions affect PRRSV infectivity in PAMs.

### The GP2a 91/97/98 aa substitutions influence PRRSV infectivity in piglets but do not influence immune responses

**Fig 9:**
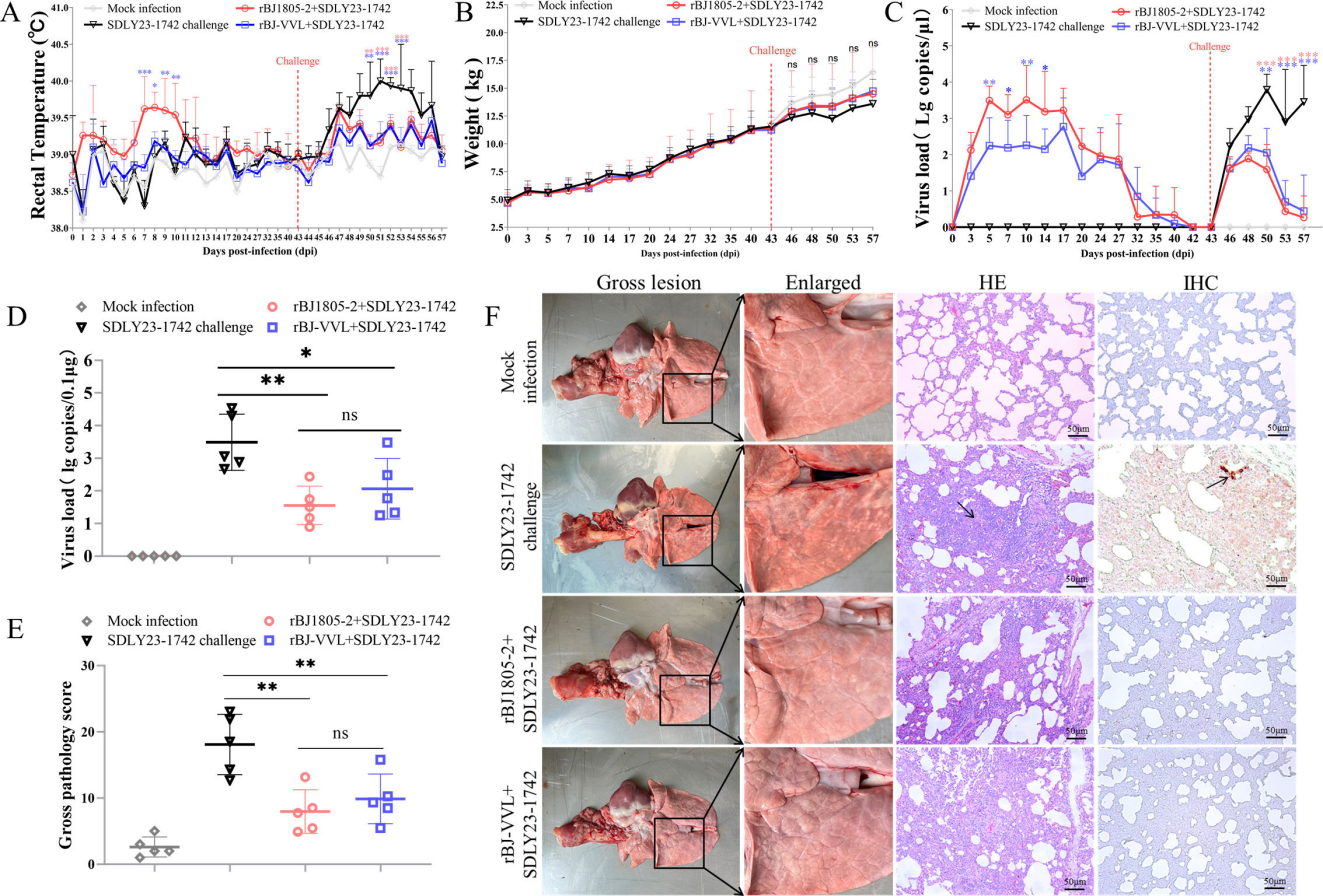
Comparison of rBJ1805-2 and rBJ-VVL inoculations in piglets. (A) Rectal temperature was determined daily for 2 weeks after rBJ1805-2 or rBJ-VVL inoculation and after the SDLY23-1742 challenge, respectively. (B) Body weight was measured weekly during serum sample collection. (C) Viremia was detected using PRRSV real-time RT-PCR assay (47). (D) The viral loads in lung samples were also detected by PRRSV real-time RT-PCR assay. (E) The gross pathology of the lungs was scored according to previously described methods (40). (F) Representative lung from each group collected at 14 dpc. Lung gross lesions in each group were enlarged. Representative pathological lesions (HE) and PRRSV-specific N antigen (IHC) in SDLY23-1742 challenged pigs were marked by black arrows.

To further investigate the influence of GP2a 91/97/98 aa substitutions on PRRSV infectivity *in vivo*, 20 PRRSV-negative 4-week-old healthy piglets were divided into four groups. Two groups of piglets were intramuscularly and intranasally inoculated with 2 mL 10^5.0^ TCID50/mL rBJ1805-2 and rBJ-VVL, respectively. Piglets in the third and fourth groups were inoculated with DMEM to serve as the negative control. The successful rBJ1805-2 and rBJ-VVL inoculations were confirmed by sequencing viruses from representative pigs at 5, 17, and 27 dpi, respectively (Fig. S3). Piglets in the rBJ1805-2-infected group developed fever (≥40°C, the highest of 40.3°C) during 7–10 dpi (*P* < 0.05), while rBJ-VVL-infected piglets did not develop fever (Fig. 9A). Both rBJ1805-2 and rBJ-VVL infections did not affect weight gain (Fig. 9B). Significantly higher viremia (*P* < 0.01) could be detected from 5 to 14 dpi in rBJ1805-2-infected piglets than in rBJ-VVL-infected piglets (Fig. 9C). These results suggested that the GP2a 91/97/98 aa substitutions also affect NADC34-like PRRSV-2 infectivity in pigs.

Both rBJ1805-2 and rBJ-VVL viremia became undetectable by 42 dpi (Fig. 9C). At 43 dpi, each piglet in the rBJ1805-2-inoculated group, rBJ-VVL-inoculated group, and the third mock-infected group was challenged with another NADC34-like SDLY23-1742 isolate (2 mL 10^4.5^ TCID50/mL), respectively. The successful challenge was confirmed by ORF5 sequencing (data not shown). Piglets in the fourth group were injected with DMEM again to serve as mock infection control. The rectal temperatures in SDLY23-1742 challenged pigs could reach ≥40°C (the highest of 40.5°C) from 50 dpi (7 dpc) to 53 dpi (10 dpc) (*P* < 0.01). Meanwhile, the rectal temperatures in rBJ1805-2 inoculated, rBJ-VVL inoculated, and mock-infected pigs were always lower than 40°C during the entire challenge period (Fig. 9A). Even though the mock-infected pigs had the highest body weight and the SDLY23-1742-challenged pigs had the lowest body weight, no statistically significant differences (*P* > 0.05) were detected among all four groups after challenge (Fig. 9B). Significantly lower viremia (*P* < 0.01) could be observed in rBJ1805−2 + SDLY23-1742 pigs (rBJ1805-2 inoculation followed by SDLY23-1742 challenge at 43 dpi) and rBJ-VVL +SDLY23-1742 pigs (rBJ-VVL inoculation followed by SDLY23-1742 challenge at 43 dpi) than in SDLY23-1742 challenged pigs from 50 dpi (7 dpc) to 57 dpi (14 dpc) (Fig. 9C). In addition, significantly lower virus loads (*P* < 0.05) were detected in lungs from rBJ1805−2 + SDLY23-1742 pigs and rBJ-VVL +SDLY23-1742 pigs than in lungs from SDLY23-1742-challenged pigs (Fig. 9D). However, no significant differences (*P* > 0.05) were observed in serum and lung samples between rBJ1805−2 + SDLY23-1742 pigs and rBJ-VVL +SDLY23-1742 pigs. Moreover, gross pathology scoring of lungs revealed that rBJ1805−2 + SDLY23-1742 pigs and rBJ-VVL +SDLY23-1742 pigs have significantly slighter pathological lesions than the SDLY23-1742-challenged pigs (*P* < 0.01) (Fig. 9E). Postmortem examination showed that severe lung consolidation could be observed in SDLY23-1742-challenged pigs while the lung lesions were mild in rBJ1805−2 + SDLY23-1742 pigs and rBJ-VVL +SDLY23-1742 pigs (Fig. 9F, gross lesion). Thickening of the alveolar septum and infiltration of lymphocytes around blood vessels could be observed in the lungs from SDLY23-1742-challenged pigs but not in the other groups of pigs during the histopathological examination (Fig. 9F, HE). PRRSV antigens could only be detected in the SDLY23-1742-challenged group but not in the other three groups (Fig. 9F, IHC). These results indicated that both rBJ1805-2 and rBJ-VVL could provide similar levels of protection against the NADC34-like SDLY23-1742 challenge.

**Fig 10:**
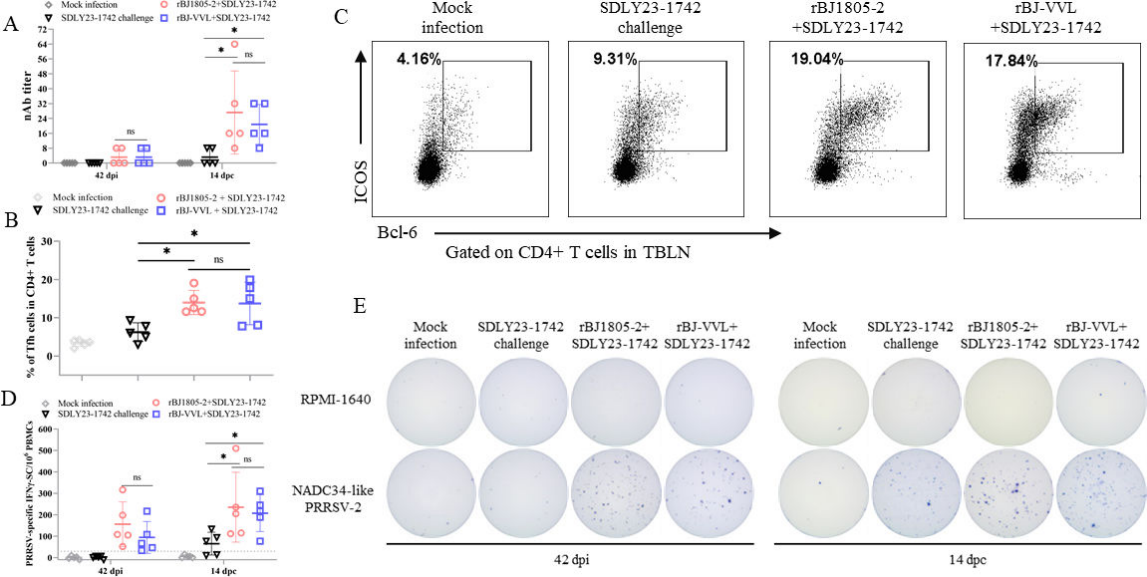
The rBJ1805-2 and rBJ-VVL strains induce similar levels of protective immune responses. (A) The nAb titers against rBJ1805-2, rBJ-VVL, and SDLY23-1742 were determined accordingly at 42 dpi and 14 dpc, respectively. (B) The percentages of porcine Tfh cells in CD4^+^ T cells in TBLNs across all groups at 14 dpc. (C) Representative dot plots depicting the percentages of porcine Tfh cells (Bcl-6 and ICOS expression) in CD4^+^ T cells in TBLNs for each group. Numbers in the quadrant represent the percentages of porcine Tfh cells. (D) Comparison of the PRRSV-specific IFN-γ-SC in PBMCs across all groups at 42 dpi and 14 dpc. The dotted line indicates the limit of detection (LOD). (E) Representative images of ELISPOT results for all groups at 42 dpi and 14 dpc.

To estimate humoral immune responses, the titers of neutralizing antibodies (nAbs) against NADC34-like PRRSV-2 isolates were determined. As shown in Fig. 10A, low levels of nAbs (titer = 1:8) were only detected in two out of five rBJ1805−2 + SDLY23-1742 pigs and rBJ-VVL +SDLY23-1742 pigs at 42 dpi. At 14 dpc, significantly increased levels of nAbs could be detected in sera from rBJ1805−2 + SDLY23-1742 pigs (1:8 ≤ titer ≤ 1:64) and rBJ-VVL +SDLY23-1742 pigs (1:8 ≤ titer ≤ 1:32) (*P* < 0.05). However, there was no statistical difference between these two groups (*P* > 0.05). T follicular helper (Tfh) cells are critical regulators for the induction of nAbs and Tfh cells in tracheobronchial lymph nodes (TBLNs) are correlated with PRRSV nAb responses (40, 48). Therefore, we also determined the percentages of Tfh cells in TBLNs by flow cytometry. Higher percentages of Tfh cells could be detected in TBLNs from rBJ1805−2 + SDLY23-1742 pigs and rBJ-VVL +SDLY23-1742 pigs than in SDLY23-1742-challenged pigs and mock-infected pigs (*P* < 0.05). But no difference was observed between rBJ1805−2 + SDLY23-1742 pigs and rBJ-VVL +SDLY23-1742 pigs (*P* > 0.05) (Fig. 10B and C), which coincided with the results of nAb responses.

To evaluate cellular immune responses, the percentages of PRRSV-specific IFN-γ-secreting cells (IFN-γ-SC) in PBMCs were also detected by ELISPOT (Fig. 10D and E). PRRSV-specific IFN-γ-SC could only be detected in rBJ1805−2 + SDLY23-1742 pigs and rBJ-VVL +SDLY23-1742 pigs but not in SDLY23-1742-challenged pigs and mock-infected pigs at 42 dpi. At 14 dpc, higher percentages of PRRSV-specific IFN-γ-SC were detected in both rBJ1805−2 + SDLY23-1742 pigs and rBJ-VVL +SDLY23-1742 pigs than in SDLY23-1742 challenged pigs (*P* < 0.05). The levels of PRRSV-specific IFN-γ-SC were not statistically different between rBJ1805−2 + SDLY23-1742 pigs and rBJ-VVL +SDLY23-1742 pigs at both 42 dpi and 14 dpc (*P* > 0.05). These results supported that rBJ1805-2 and rBJ-VVL induce similar levels of cellular immunity. The above findings suggested that the GP2a 91/97/98 aa substitutions do not significantly influence immune responses.

## DISCUSSION

The high genetic diversity of PRRSV isolates complicates the prevention and control of diseases in pigs, which might even increase the risk of cross-species transmission. A recent study has shown that simian arterivirus (simian hemorrhagic fever virus, SHFV) is poised for spillover to humans (29). PRRSV is an arterivirus with a restricted host and cell tropism. The pig is the only natural host for PRRSV. Marc-145 cells derived from monkey kidney are one of the few cell lines that may support PRRSV replication *in vitro*. A numerous of studies showed that currently prevalent PRRSV isolates frequently fail to infect Marc-145 cells (40, 46, 49, 50). Therefore, this study started with exploring the exact determinant of PRRSV adaptation to Marc-145 cells. Our results demonstrated for the first time that the GP2a 91/97/98 aa substitutions are a sufficient and necessary determinant of Marc-145 adaptation in lineage 1 PRRSV-2 isolates, a sufficient but not necessary determinant in HP-PRRSV-2, and a necessary but not sufficient determinant in PRRSV-1, respectively. In addition, the GP2a 91/97/98 aa substitutions affected PRRSV infectivity in PAMs and piglets but did not influence immune responses. These findings not only decipher an exact determinant of PRRSV tropism and infectivity but also have important implications in PRRS vaccine development.

Marc-145 cells play an important role in PRRSV isolation and vaccine development (15, 51). However, failure to infect Marc-145 cells was frequently reported in lineage 1 PRRSV-2 (NADC34-like and NADC30-like) isolates in recent years (46, 49, 50). The 5′ UTR and 3′ UTR are not responsible for NADC34-like PRRSV-2 adaptation on Marc-145 cells (49). Minor envelope proteins are a major determinant of arterivirus tropism (16) and GP2a-GP3 is a main determinant of PRRSV-2 tropism to Marc-145 cells (34). More specifically, the 98 and 160 residues in GP2a were reported as key determinants of PRRSV-2 tropism to Marc-145 cells (35, 36). Meanwhile, the GP2a 88/95 aa is critical to PRRSV-1 growth in CL2621 cells (32). The 88/94/95 aa in GP2a of PRRSV-1 is essential for Marc-145 adaptation (33). However, these previous studies generally explored the key determinants of Marc-145 adaptation based on only one specific PRRSV strain, and their results were somehow controversial. Therefore, a comprehensive evaluation was executed in this study, and the 91/97/98 aa substitutions in GP2a were identified as a critical determinant of Marc-145 tropism for distinct PRRSV strains. For lineage 1 PRRSV-2 isolates, they are a sufficient and necessary determinant. For HP-PRRSV-2 strain, they are also sufficient but not necessary. GP2a-GP3 (excluding GP2a 91/97/98 aa substitutions and GP3-GP4 overlap region) and GP3-GP4 regions contain two additional sufficient determinants. For PRRSV-1 isolates, they are necessary but not sufficient. The GP3-GP4 overlap region is also needed to provide Marc-145 adaptation for PRRSV-1. Noticeably, the rXTB-M23-TMF and rXTB-M34 chimeric PRRSV-2 viruses (Fig. 5) and the rHTA-FIL-3 chimeric PRRSV-1 virus (Fig. 6) are adaptive to Marc-145 cells, reflecting the importance of GP3 in PRRSV tropism, which deserves further investigation. In addition, major envelope proteins (GP5 and M) and nsp2 have also been reported to affect PRRSV tropism (24, 37, 38). Our results showed that GP5-M did not change HP-PRRSV-2 tropism (Fig. 4A-III and B-III). The nsp2 replaced chimeric virus (rXTB-TMF-nsp2) could not be rescued, which is consistent with a previously reported CHsx1401-nsp2_JX_ strain likely due to a compatibility issue between lineage 8 HP-PRRSV-2 nsp2 and lineage 1 PRRSV-2 structural proteins (52). Overall, the systematic evaluations in this study deciphered that the 91/97/98 aa substitutions in GP2a play critical roles in determining PRRSV tropism to Marc-145 cells.

More importantly, the GP2a 91/97/98 aa substitutions also affected PRRSV infectivity in PAMs and piglets. A recent study showed that the single GP2a 98th mutation did not impact PRRSV-2 replication in PAMs and did not affect the infectivity and pathogenicity in piglets (36). However, the GP2a K160I substitution could reduce PRRSV infectivity in PAMs and pigs (35). In this study, we validated that the GP2a 91/97/98 aa substitutions are responsible for the distinct PRRSV infectivity in PAMs. In addition, the GP2a 91/97/98 aa substitutions could also influence PRRSV infectivity in piglets. These results indicated that the GP2a 91/97/98 aa substitutions not only are a critical determinant for PRRSV tropism but also play an important role in changing PRRSV infectivity in natural hosts (Fig. 8 and 9C).

**Fig 11:**
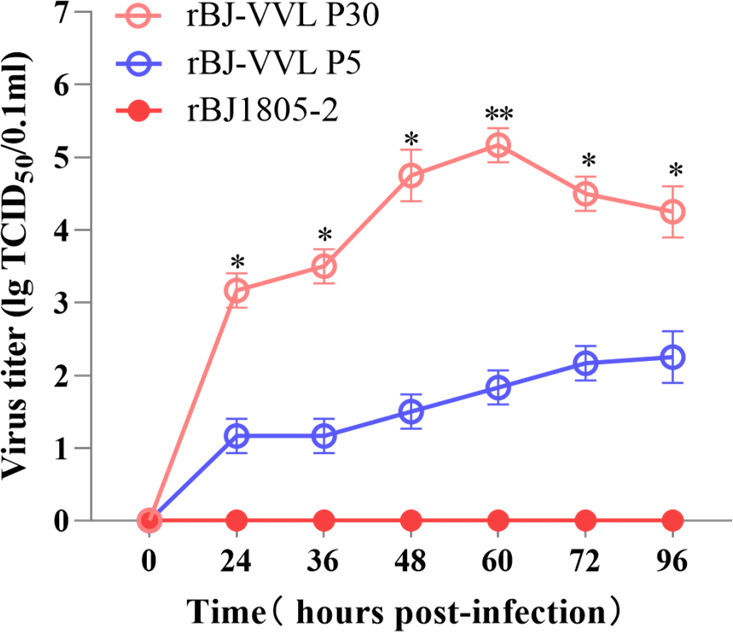
Comparison of replication efficacies among rBJ1805-2, rBJ-VVL-P5, and rBJ-VVL-P30 in Marc-145 cells. Virus titration was performed using supernatants collected at 0, 24, 36, 48, 72, and 96 hpi. The viral titer used for infection is 0.01 MOI.

Vaccination is a widely utilized and effective strategy to control PRRS. However, PRRS-modified live vaccines (MLVs) only provide satisfied homologous protection but cannot confer sufficient heterologous protection. Lineage 1 PRRSV-2 isolates are predominant in China, but commercial PRRS MLVs confer limited cross-protection against these isolates (53, 54). Therefore, developing effective vaccines specifically against lineage 1 PRRSV-2 isolates will be of great significance for PRRS control. The obtained rBJ-VVL strain seems to be an eligible candidate strain for lineage 1 PRRSV-2 strain-specific vaccine development. The reasons are as follows: (i) The rBJ-VVL strain is permissive to Marc-145 cells. In addition, after 30 times of passaging in Marc-145 cells, the GP2a 91/97/98 aa substitutions are stable *in vitro,* and the rBJ-VVL-P30 can reach a significantly higher titer than the rBJ-VVL-P5 (*P* < 0.05) (Fig. 11). Complete genome sequencing identified 13 aa substitutions between rBJ-VVL-P5 and rBJ-VVL-P30 (Table 1), which might contribute to the enhanced replication efficacy and deserve further clarification. Moreover, sequencing viruses from rBJ-VVL inoculated pigs from 5 to 27 dpi also suggested that the GP2a 91/97/98 aa substitutions may be stable *in vivo* (Fig. S3). These features will facilitate the development of PRRS vaccines. (ii) Even though rBJ-VVL inoculation did not cause fever while the parental rBJ1805-2 did (Fig. 9A), it is hasty to assert that the GP2a 91/97/98 aa substitutions could significantly decrease the pathogenicity, especially considering that nsp9 and nsp10 have been identified as major determinants of PRRSV virulence (55–57). However, the parental rBJ1805-2 virus is mildly pathogenic to pigs. In addition, the rBJ-VVL strain has even lower infectivity than the parental rBJ1805-2 virus in piglets. Therefore, no serious safety issues were noticed yet to consider rBJ-VVL as a candidate vaccine strain. (iii) Both rBJ-VVL and its parental rBJ1805-2 strains can induce similar levels of immune responses and provide satisfactory protection against the NADC34-like PRRSV-2 challenge (Fig. 10), indicating that the rBJ-VVL strain can provide sufficient homologous protection against NADC34-like isolates.

**TABLE 1:** Comparison of substitutions among rBJ-VVL-P5, rBJ-VVL-P30, representative wild-type isolates, and vaccine strains

No.	Viral protein	site	rBJ-VVL		IA/2014/NADC34	JS2021NADC34	NADC30	SD17-38	CH-1a		JXA1		VR2332	
			P5	P30	Wild	Wild	Wild	Wild	Wild	CH-1R^*a*^	Wild	P80^*a*^	Wild	Resp MLV^*a*^
1	nsp2	318	A	T	T	T	T	A	T	T	T	T	T	T
2		373	N	S	N	N	N	N	N	N	N	N	D	D
3		599	I	T	I	I	I	I	A	A	A	A	V	V
4	nsp4	187	Y	C	H	H	S	S	C	C	C	C	S	S
5	nsp5	30	V	G	V	F	G	G	G	G	G	G	S	S
6		421	Y	C	Y	Y	Y	H	H	H	H	H	H	H
7	nsp10	313	Y	H	Y	Y	Y	Y	Y	Y	Y	Y	Y	Y
8	GP2a	132	S	N	S	S	S	S	S	S	S	S	S	S
9	GP4	103	L	F	L	L	L	L	L	L	L	L	L	L
10	GP5a	3	R	G	R	R	R	R	K	K	K	K	K	K
11		42	F	I	F	F	S	F	F	I	Y	Y	S	S
12	GP5	38	H	Q	H	H	H	H	H	Q	H	H	H	H
13		167	I	V	V	V	V	V	V	V	V	V	V	V

^*a*^ The symbols represent the corresponding vaccine strains.

Previous studies have identified that B-cell epitopes are located in 36–51 aa, 117–139 aa, and 120–142 aa of GP2a in PRRSV-1, and within 41–55 aa and 121–135 aa in GP2a of PRRSV-2, respectively (58, 59). Meanwhile, the GP2a 89–100 peptide has low reactivity with PRRSV antisera (only 1 out of 17) (60). In addition, it has been demonstrated that GP2a protein is not the predominant antigen for T-cell response (61). No PRRSV-specific T-cell epitope has been identified in GP2a so far (62–67). These studies supported that the GP2a 91/97/98 aa are not located in the major B-cell and T-cell epitopes, which may account for the unchanged immune responses.

This study still has several limitations. At first, bioinformatics analysis showed that there are more than 40 GP2a 91/97/98 aa patterns among PRRSV isolates (Fig. S2). This study mainly evaluated the influences of four patterns (“TMF” and “VVL” in PRRSV-2, “IMF” and “FIL” in PRRSV-1) on cellular tropism and viral infectivity. However, the influences of other patterns on tropism, infectivity, and even cross-species transmission deserve further investigation. A more significant limitation is that this study did not clarify how the GP2a 91/97/98 aa substitutions affect virus-cell interactions. Previous studies showed that PRRSV GP2a and GP4 can interact with CD163 receptors to mediate PRRSV entry (27, 68). Our preliminary data indicated that the GP2a 91/97/98 aa substitutions do not influence PRRSV attachment and internalization but may affect viral uncoating and replication (Fig. S5), which are consistent with previous studies that the interaction between GP2a-GP4 and CD163-SRCR5 is essential to initiate PRRSV uncoating and replication (27, 69). Therefore, it is rational to hypothesize that the GP2a 91/97/98 aa substitutions might affect its binding to distinct CD163. However, our preliminary data also suggested that the GP2a 91/97/98 aa substitutions do not influence the bindings between GP2a (from rBJ1805-2 and rBJ-VVL) and SRCR5 (from porcine CD163 and monkey CD163) (Fig. S6). A recent study showed that mutated CD163 proteins that resisted PRRSV infection can still bind to viral glycoprotein, suggesting that PRRSV envelope proteins may form multiple interactions with CD163, or the CD163 regions important for infection have other cellular binding partners required for PRRSV infection (70). Similarly, another study indicated that intracellular processing of arterivirus glycoproteins may be required before CD163 receptor binding can occur (29). Anyway, more efforts must be made to clarify the underlying mechanisms.

Overall, this study demonstrates for the first time that GP2a 91/97/98 aa substitutions play critical roles in determining PRRSV adaptation to Marc-145 cells. Moreover, the GP2a 91/97/98 aa substitutions significantly influence PRRSV infectivity in PAMs and piglets but do not affect immune responses. These findings not only provide new insights into PRRSV tropism and infectivity but also have guiding significance for PRRS vaccine development.

## MATERIALS AND METHODS

### Viruses, cells, and antibodies

Five Chinese PRRSV isolates were used in this study. The evolutionary relationships among them are shown in Fig. S4. BJ1805-2 strain (GenBank accession no. PQ373814) is a NADC34-like PRRSV-2 isolated from Beijing in 2018 (.^71^). SD17-38 strain is a NADC30-like PRRSV-2 isolated from Shandong province in 2017 (31). XJ17-5 strain is an HP-PRRSV-2 isolated from Xinjiang Uygur Autonomous Region in 2017 (43). HLJB1 strain is a PRRSV-1 isolated from Heilongjiang Province in 2014 (72). SDLY23-1742 strain is a NADC34-like PRRSV-2 isolated from Shandong province in 2023 (PQ373813) (71). PAMs were harvested from the lung lavage fluid of 6-week-old PRRSV-free piglets as previously described (8). PAMs were cultured in Roswell Park Memorial Institute 1640 medium (RPMI-1640) (HyClone, USA) supplemented with 10% fetal bovine serum (FBS) (EallBio, China), 100 U/mL penicillin, and 100 µg/mL streptomycin (Solarbio, China). Marc-145 cells were obtained from three different laboratories for Marc-145 adaptation evaluation. BHK-21 cells stored in our laboratory were used for the transfection of chimeric plasmids. Marc-145 and BHK-21 cells were cultured in Dulbecco minimum essential medium (DMEM) (HyClone, USA) supplemented with 10% FBS and antibiotics. PRRSV N-specific murine mAb 15A1 was used for PRRSV-1 and PRRSV-2 detection (1:500 dilution in IFA, 1:1,000 dilution in WB) (10, 73).

### Construction of NADC34-like and NADC30-like PRRSV-2 cDNA clones

To construct NADC34-like PRRSV-2 rBJ1805-2, a stuffer fragment (900 bp) containing the cytomegalovirus (CMV) promoter, four restriction enzyme sites (*PacI*, *Bsu36I*, *XbaI*, and *AscI*), and bovine growth hormone (BGH) polyadenylation signal was synthesized and inserted after the BamHI restriction site of the low-copy-number pACYC177 plasmid (Suzhou Genewiz company). The BJ1805-2 viral RNA was extracted with TRIpure reagent (Aidlab, Beijing, China), and then reverse-transcribed to generate cDNA using the HiScript III 1st Strand cDNA Synthesis Kit (+gDNA wiper, Vazyme, Nanjing, China). The BJ1805-2 genome was divided into three fragments (F1, F2, and F3). Each fragment was amplified with primer pairs shown in Table S1. The plasmid pACYC177 was linearized by double digestion with corresponding restriction enzymes. Each fragment was inserted into the linearized pACYC177 backbone step-by-step using T4 DNA ligase (Invitrogen, USA). The entire pACYC177-rBJ1805-2 cDNA clone was confirmed by Sanger sequencing and named rBJ1805-2. For NADC30-like PRRSV-2 rSD17-38 construction, the SD17-38 genome was divided into four segments (F1, F2, F3, and F4) using five restriction enzyme sites (*PacI*, *ScaI*, *NotI*, *Bsp1407I*, and *AscI*). The pACYC177-rSD17-38 cDNA clone was constructed using similar strategies as described above, which was also confirmed by Sanger sequencing and denominated as rSD17-38. PRRSV-1 rHLJB1 and HP-PRRSV-2 rXJ17-5 clones have been constructed previously (40, 74).

### Construction of chimeric PRRSV cDNA clones

Based on the above four PRRSV cDNA clones, four chimeric viruses (rBTX234, rXTB234, rSTX234, and rHTA23) switching minor envelope protein-encoding genes were first constructed to facilitate the subsequent constructions of fragment replacements. The rBJ1805-2 clone was amplified with two primer pairs (rBJ-XbaI-F3 + rBTX234-ORF1b-R and rBTX234-ORF5-F + rBJ-NOT1-2fu-1) to produce rBTX234-1 and rBTX234-3 fragments. Meanwhile, the rXJ17-5 clone was amplified with a primer pair (rBTX234-ORF2-F + rBTX234-ORF4-R) to produce the rBTX234-2 fragment. Then, these three fragments (combined into an F3 fragment) were ligated with pACYC177-rBJ1805-2-F1+F2 by seamless cloning using ClonExpress Ultra One Step Cloning Kit (Vazyme, Nanjing, China) to generate the chimeric clone of rBTX234 (a chimeric rBJ1805-2 virus containing ORFs 2–4 from rXJ17-5). Similarly, three primer pairs (rXTB234-ORF2-F + rXTB234-ORF4-R, rXJ17-5-AscI-F3-F1 + rXTB234-ORF4-R, and rXTB234-ORF4-F + rBJ-NOT1-2fu-1), (rSD17-38-F3 + rSTX234-ORF1b-R, rSTX234-ORF2-F + rSTX234-ORF4-R, and rSTX234-ORF5-F + rBJ-NOT1-2fu-1), and (HLJB1-BGLII-F3 + rHTA23-ORF1b-R, rHTA23-ORF2-F + rHTA23-ORF3-R, and rHTA23-ORF3-F + rBJ-NOT1-2fu-1) were used to generate rXTB234 (a chimeric rXJ17-5 virus containing ORFs 2–4 from rBJ1805-2), rSTX234 (a chimeric rSD17-38 virus containing ORFs 2–4 from rXJ17-5), and rHTA23 (a chimeric rHLJB1 virus containing ORFs 2–3 from Amervac vaccine strain), respectively.

Based on the above four PRRSV cDNA clones and four chimeric cDNA clones, another 55 chimeric cDNA clones were constructed as shown in Table S2. Each modified segment of these chimeric cDNA clones was generated by two amplicons (1st and 2nd). The 1st and 2nd amplicons were seamlessly cloned into corresponding linearized vectors to obtain chimeric plasmids.

To construct a chimeric virus switching major envelope protein-encoding genes (ORFs 5–6), the rXJ17-5-TMF clone was amplified with a primer pair (rXJ17-5-AscI-F + rXTB-TMF-56-ORF4-R) to produce the rXTB-TMF-56–1 fragment. The rBJ1805-2 clone was amplified with a primer pair (rXTB-TMF-56-ORF5-F + rXTB-TMF-56-ORF6-R) to produce the rXTB-TMF-56–2 fragment. The rXJ17-5 clone was amplified with a primer pair (rXTB-TMF-56-ORF7-F + rBJ-NOT1-2fu-1) to produce rXTB-TMF-56–3. Then, these three fragments were seamlessly cloned into the pACYC177-rXJ17-5-F1+F2 linearized vector to obtain rXTB-TMF-56.

To construct a chimeric virus switching nsp2, the rXJ17-5 was amplified with primer pairs (pACYC177-PAC1-fusion-F + rXTB-nsp1-R and rXTB-nsp3-F + XJ17-5-AflII-fusion-R) to produce rXTB-nsp2-TMF-1 and rXTB-nsp2-TMF-3, respectively. The rBJ1805-2 was amplified with a primer pair (rXTB-nsp2-F + rXTB-nsp2-R) to generate rXTB-nsp2-TMF-2. The three fragments were seamlessly ligated with the pACYC177-rXJ17-5-TMF-F2+F3 vector to obtain rXTB-nsp2-TMF.

### Rescue of viruses

BHK-21 cells were seeded into 12-well cell culture plates at the concentration of 1 × 10^5^ cells per well. When the cell density reached 90%, each full-length cDNA clone was transfected into the cells with Lipofectamine 3000 (Invitrogen, USA) (10, 73). After 48 hours post-transfection, the cells and supernatant were freeze-thawed three times and then centrifuged at 5,000 rpm for 5 min. The supernatant was collected and stored at −80℃. PAMs were seeded into 12-well plates at 1 × 10^6^ cells per well and infected by 500 μL filtered supernatant at 24 hours post-seeding. After virus adsorption for 2 h, the supernatant was discarded, gently washed twice with PBS, and a fresh RPMI-1640 medium containing 2% FBS was added. The supernatant was harvested daily to monitor the replication of the rescued virus. The infected cells were detected by IFA to confirm the successful rescue of each virus.

Marc-145 cells were inoculated into 12-well plates as 1.5 × 10^5^ cells per well. When the cell density reaches 90%, it is infected by the supernatant from infected PAMs for up to 7 days. The cytopathic effect (CPE) was monitored daily. IFA was also used to detect infected Marc-145 cells to determine whether the chimeric virus obtained Marc-145 cell tropism. Each Marc-145 adaptive chimeric virus was serially passaged in Marc-145 cells five times.

### Indirect immunofluorescence assay

The PAMs and Marc-145 cells were fixed with 4% paraformaldehyde for 30 min, permeabilized by 0.^5^% TritonX-100, and then blocked with 5% BSA. The PRRSV N-specific murine mAb ^15^A1 (1:500 dilution) was used as the primary antibody, while DyLight ^594^ (goat anti-mouse IgG, 1:1,000, Invitrogen) was used as the secondary antibody. The nucleus was stained with a DAPI staining solution (0.^5^ μg/mL, Beyotime, Shanghai, China) for 15 min. Then, confocal microscopy (Leica SP8, Germany) was utilized to evaluate the successful rescues of chimeric viruses (40).

### Viral plaque assay

Marc-145 cells were seeded into 24-well plates at 1 × 10^5^ cells per well and cultured in an incubator with 5% CO_2_ at 37 °C until 100% confluent monolayer cells. Then 10-fold serially diluted viruses were used to infect Marc-145 cells. After 2 hours of adsorption, the supernatant was discarded, and cells were gently washed with 1×PBS three times. In addition, the infected cells were coated with 2% FBS methylcellulose solution (50 mL medium formula: 23.5 mL DMEM + 1 mL FBS + 500 μL penicillin streptomycin + 25 mL 2% methylcellulose). The 24-well plates were cultured in 5% CO_2_ at 37℃ for 6 days. Subsequently, the methylcellulose covering solution was discarded, 4% paraformaldehyde was added to fix overnight, and crystal violet dyeing solution was added for 1 h (10, 73). The plaque shape was recorded after washing three times with PBS.

### Viral titration

The growth curves of Marc-145 adapted chimeric viruses were measured as previously described (72). The growth of rXJ17-5 in Marc-145 cells was set as a positive control. Each virus at 0.01 multiplicity of infection (MOI) was used for inoculation. The supernatants were collected at 0, 12, 24, 36, 48, 72, and 96 hpi and stored at −80℃ until use. Marc-145 cells were seeded into 96-well plates at 3×10^4^ cells per well. DMEM medium containing 10% FBS was used for Marc-145 cell culture in an incubator at 37°C with 5% CO_2_. When reached 80% confluent monolayer cells, each virus was added into 96-well plates with eight replicates. After virus adsorption for 2 h, the infected cells were washed twice with 1× PBS and the supernatant was replaced with 200μL fresh DMEM medium containing 2% FBS. At 7 days post-infection (dpi), the wells presenting CPE were recorded and the viral titers were calculated using the Reed-Muench method (16).

### Western blotting

WB was performed as we previously described (74). In detail, cells were first lysed in radio-immunoprecipitation assay (RIPA) buffer (50 mM Tris pH 7.2, 150 mM NaCl, 1% sodium deoxycholate, 1% Triton X-100). And then the extracted proteins were separated by 15% SDS-PAGE gels and transferred to polyvinylidene fluoride (PVDF) membranes (Merck Millipore, USA). Membranes were blocked for 1 h using 5% non-fat milk in Tris-buffered saline containing 0.1% Tween-20 (TBST). Subsequently, the membrane was incubated with the primary mAbs (1:1,000 anti-β-actin and 1:1,000 PRRSV-N mAbs 15A1) in a blocking buffer at 4℃ overnight. Later on, the membrane was incubated with HRP-conjugated Goat Anti-Mouse IgG (1:10,000, BBI, Shanghai, China) at 37°C for 1 h. Upon the addition of an enhanced chemiluminescence (ECL) substrate (Biosharp, Beijing, China), the protein signals were visualized and captured by the WB imaging system (Tanon, Shanghai, China).

### Animal experiments

Twenty 4-week-old PRRSV-free piglets were divided into four groups (five pigs per group) and utilized for the inoculation and challenge study. Piglets in the first two groups were inoculated with rBJ1805-2 and rBJ-VVL (2 mL, 10^5.0^ TCID50/mL, 5th passage), respectively. Piglets in the third and fourth groups were inoculated with DMEM to serve as the negative control. At 43 dpi, piglets in groups 1, 2, and 3 were challenged with NADC34-like SDLY23-1742 isolate (2 mL, 10^4.5^ TCID50/mL, 3rd passage), while piglets in group 4 were inoculated with DMEM again to serve as the mock infection control. The rectal temperature and clinical symptoms were monitored daily after inoculation and challenge. Body weight was also determined weekly. Serum samples were acquired weekly for viremia detection. The virus loads in serum and lung samples were analyzed by PRRSV real-time RT-PCR assay (74). All survived pigs were euthanized at 14 dpc, and tissue samples were collected for histopathological and immunohistochemical examinations (9).

### Virus neutralization test

The sera collected at 42 dpi and 14 dpc were used for VNT against rBJ1805-2, rBJ-VVL, and SDLY23-1742 isolates, respectively. VNT was performed as previously described (75). Briefly, serum dilutions were incubated with 200 TCID50 of virus for 1 h, and then the well contents were transferred to PAMs or Marc-145 cells and incubated for up to 7 days. The absence of CPE at a 1:8 dilution was considered positive for the presence of virus-neutralizing activity.

### Flow cytometry

Flow cytometry (FCM) analysis was performed to detect follicular helper T (Tfh) cells as we previously described (40, 76). Briefly, tracheobronchial lymph nodes (TBLNs) were dissected and scrubbed through a 70 µm cell strainer, followed by density gradient centrifugation using Ficoll-Paque (TBD science, China). The mononuclear cells at the interface layer were collected and then counted using a hemocytometer (Sigma, UK). Subsequently, the cells (2 × 10^6^) were first stained with Fixable vital dye eFluor 780 (Thermo Fisher Scientific, USA), followed by staining with a cocktail of fluorochrome-labeled mAbs against cell surface markers CD3 (clone BB23-8E6-8C8, BD), CD4 (clone 74–12-4, BD), and anti-mouse ICOS (clone C398.4A, Biolegend). After surface staining, cells were fixed and permeabilized using eBioscience Foxp3/Transcription Factor Staining Buffer Set (Thermo Fisher Scientific, USA), and then stained with anti-human Bcl-6-PE mAb (clone K112-91, BD). Finally, the stained cells were resuspended in PBS solution for FCM analysis. Data were acquired on a CytoFLEX S flow cytometer (Beckman Coulter, USA) and analyzed using FlowJo software (Tree Star Inc., Ashland, OR). The gating strategy employed to quantify frequencies of Tfh cells was described previously (76).

### IFN-γ-ELISPOT

Porcine IFN-γ-ELISPOT assays were modified from a previous study (77). Briefly, MultiScreen-IP plates (Millipore, USA) were coated with 10 µg/mL of anti-porcine IFN-γ mAb (pIFN-γ-I, Mabtech, Sweden) overnight at 4 °C, and then blocked with the complete RPMI-1640 medium for 2 hours at 37 °C. Peripheral blood mononuclear cells (PBMCs) were isolated from heparin-anticoagulated blood by density gradient centrifugation using Ficoll-Paque (TBD science, China). Subsequently, PBMCs were seeded in duplicate into prepared ELISPOT plates at a density of 3 × 10^5^ cells/well and stimulated with NADC34-like SDLY23-1742 isolate at an MOI of 0.1 or complete RPMI-1640 medium alone (blank control). After 24 h incubation, the plates were washed and incubated with biotinylated anti-porcine IFN-γ mAb (0.5 µg/mL, P2C11, Mabtech, Sweden) for 2 h, followed by adding streptavidin-HRP (1:1,000, Mabtech, Sweden). Spots were developed with TMB ELISpot substrate (Mabtech, Sweden) and quantitated using an S6-Entry ImmunoSpot Analyzer (CTL, USA). Results were expressed as the average number of IFN-γ-secreting cells (IFN-γ-SC) per million PBMCs, with the background (blank control) subtracted. The limit of detection (LOD) was determined at 30 IFN-γ-SC/10^6^ PBMCs.

### Statistical analysis

The rectal temperature, body weight, and viremia were presented at means ± standard deviations (SDs). One-way ANOVA and two-way ANOVA in the GraphPad Prism 8.0.3 project were used for differential analyses among groups (10, 73). The statistical significance was set at *P* < 0.05.

## Data Availability

The data underlying this article are available in this article and its supplemental material.

## SUPPLEMENTAL MATERIAL

Supplemental material jvi.00048-25-s0001.pdf Figures S1 to S6; Tables S1 and S2.

ASM does not own the copyrights to Supplemental Material that may be linked to, or accessed through, an article. The authors have granted ASM a non-exclusive, world-wide license to publish the Supplemental Material files. Please contact the corresponding author directly for reuse.
