# Supplementary material for: The GP2a 91/97/98 amino acid substitutions play critical roles in determining PRRSV tropism and infectivity but do not affect immune responses
Source: J Virol. 2025 Mar 12;99(4):e00048-25. doi: 10.1128/jvi.00048-25 (PMC11998492; doi:10.1128/jvi.00048-25)
Supplement: Supplemental material — Figures S1 to S6; Tables S1 and S2. [file jvi.00048-25-s0001.pdf]

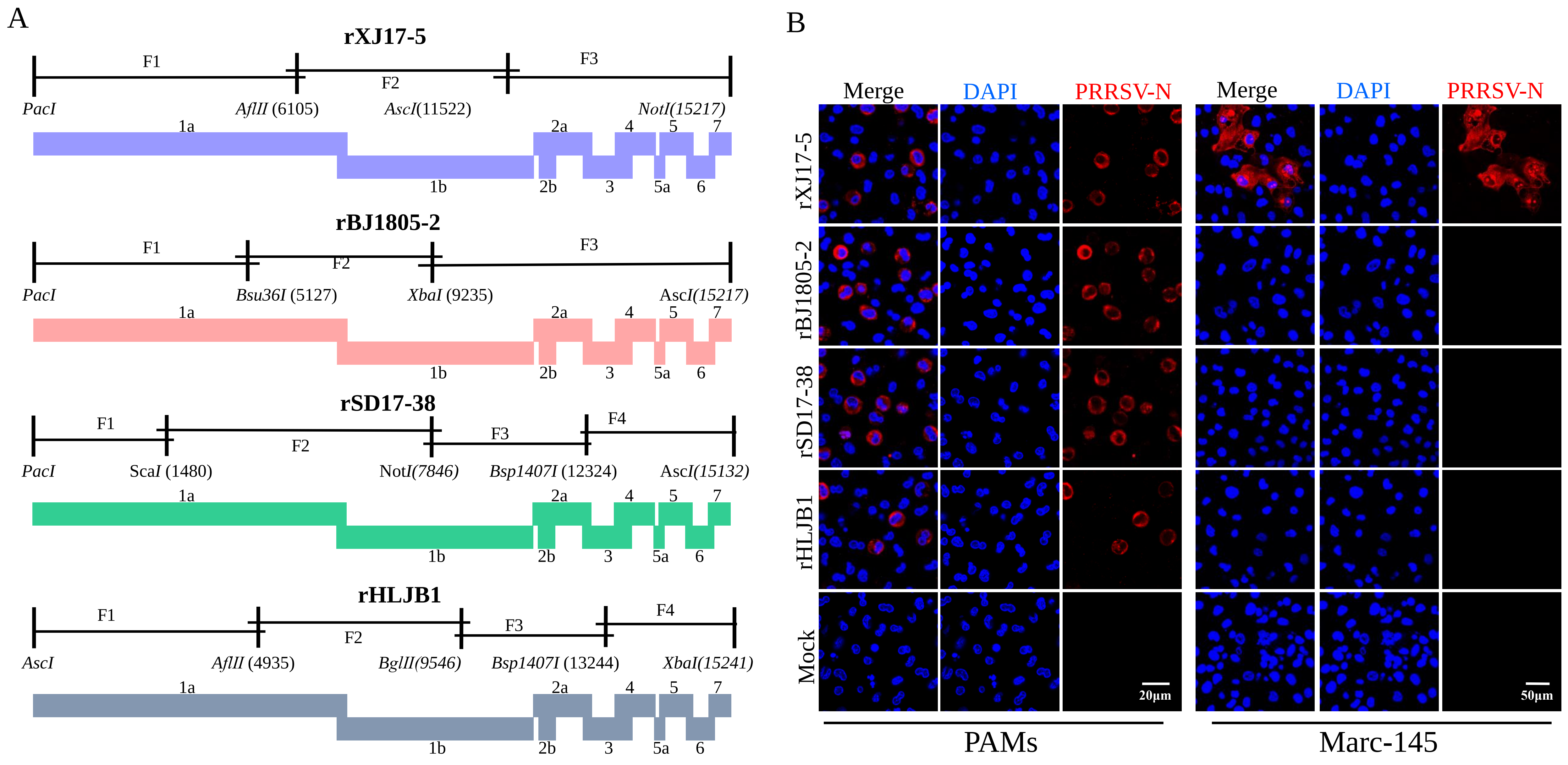

FIG S1 Construction of four PRRSV infectious clones. (A) Construction strategies for HP-PRRSV-2 rXJ17-5, NADC34-like PRRSV-2 rBJ1805-2, NADC30-like PRRSV-2 rSD17-38, and PRRSV-1 rHLJB1 infectious clones. The restriction sites used for each clone were shown. (B) IFA detection in infected PAMs and Marc-145 cells was used to confirm the successful rescues of corresponding viruses. The rXJ17-5 is adaptive to Marc-145 cells, while the other three rescued viruses are not adaptive to Marc-145 cells.

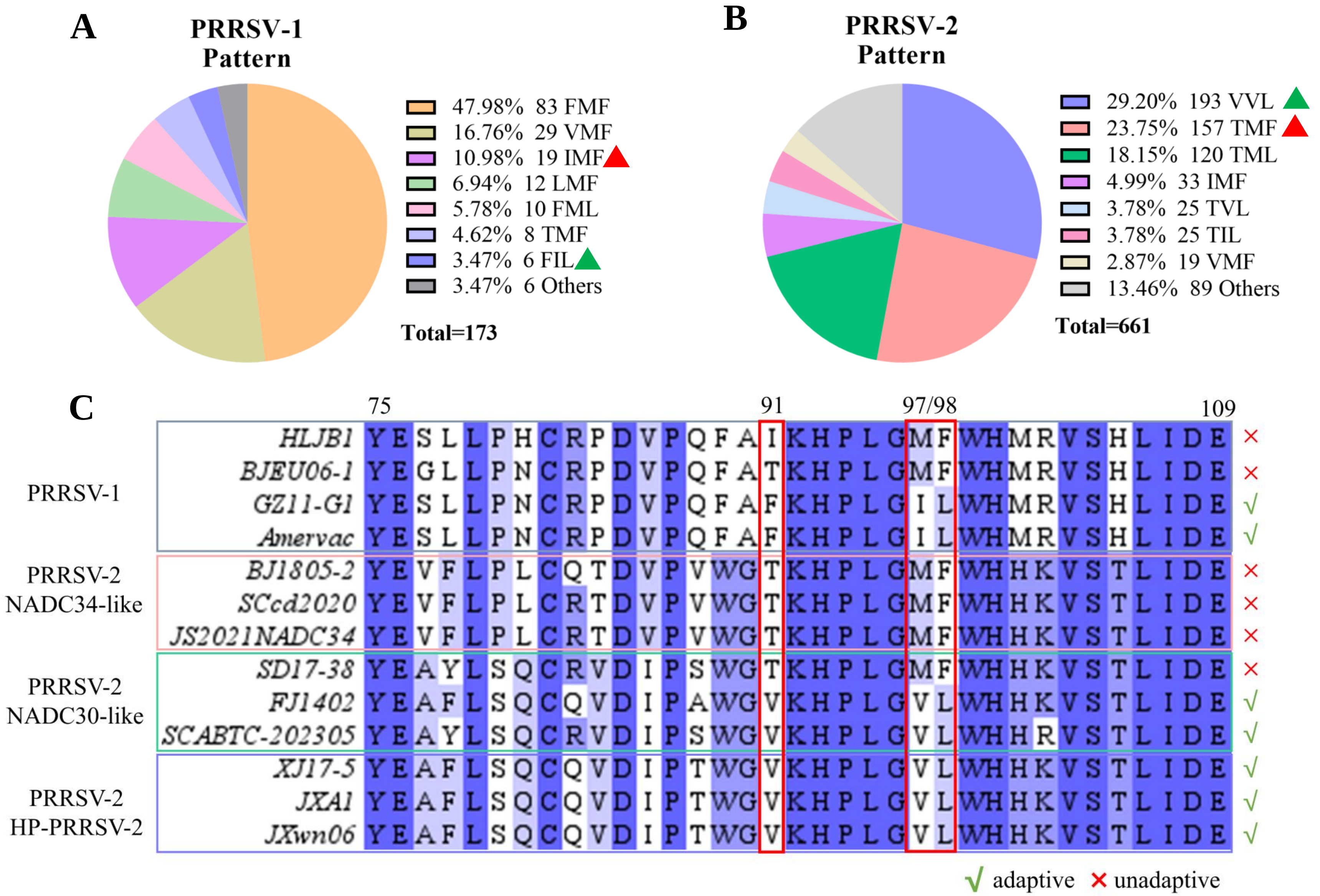

FIG S2 GP2a 91/97/98 aa patterns in PRRSV-1 and PRRSV-2 isolates. (A-B) The percentages of GP2a substitution patterns in 173 PRRSV-1 and 661 PRRSV-2, respectively. The influences of two patterns ("IMF" and "FIL") in PRRSV-1 and two patterns ("VVL" and "TMF") in PRRSV-2 on Marc-145 cell tropism have been evaluated in this study. These four patterns are highlighted with color triangles. (C) Comparison of GP2a substitution patterns among representative isolates. The Marc-145 cell tropisms of the representative PRRSV isolates shown here have been determined previously, which are exactly consistent with our chimeric virus results.

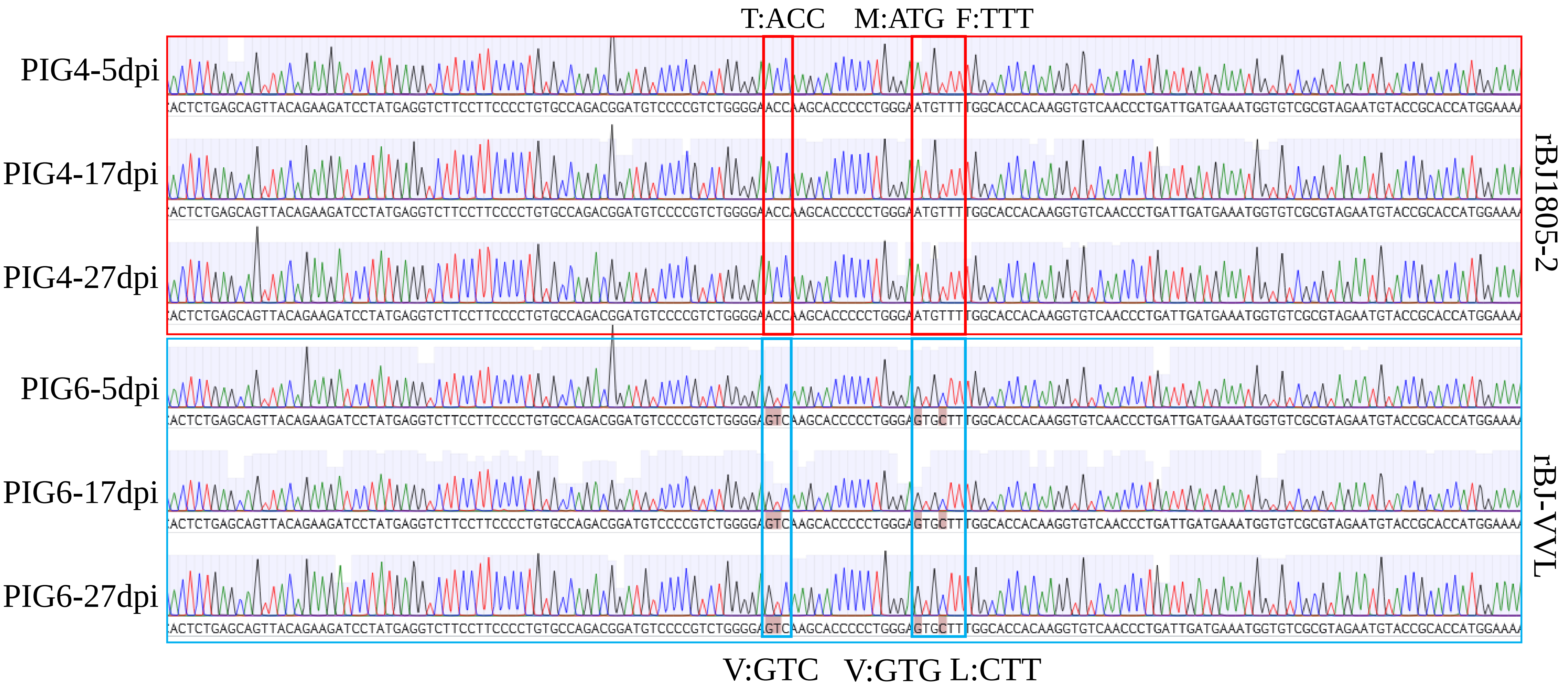

FIG S3 The stability of GP2a 91/97/98 aa substitutions *in vivo* was evaluated. The viruses were amplified from representative pigs (rBJ1805-2 infected pig 4 and rBJ-VVL infected pig 6) at 5, 17, and 27 dpi. The amplicons were sent out for ORF2 sequencing. The GP2a 91/97/98 aa encoding sequences from each pig are identical from 5 dpi to 27 dpi.

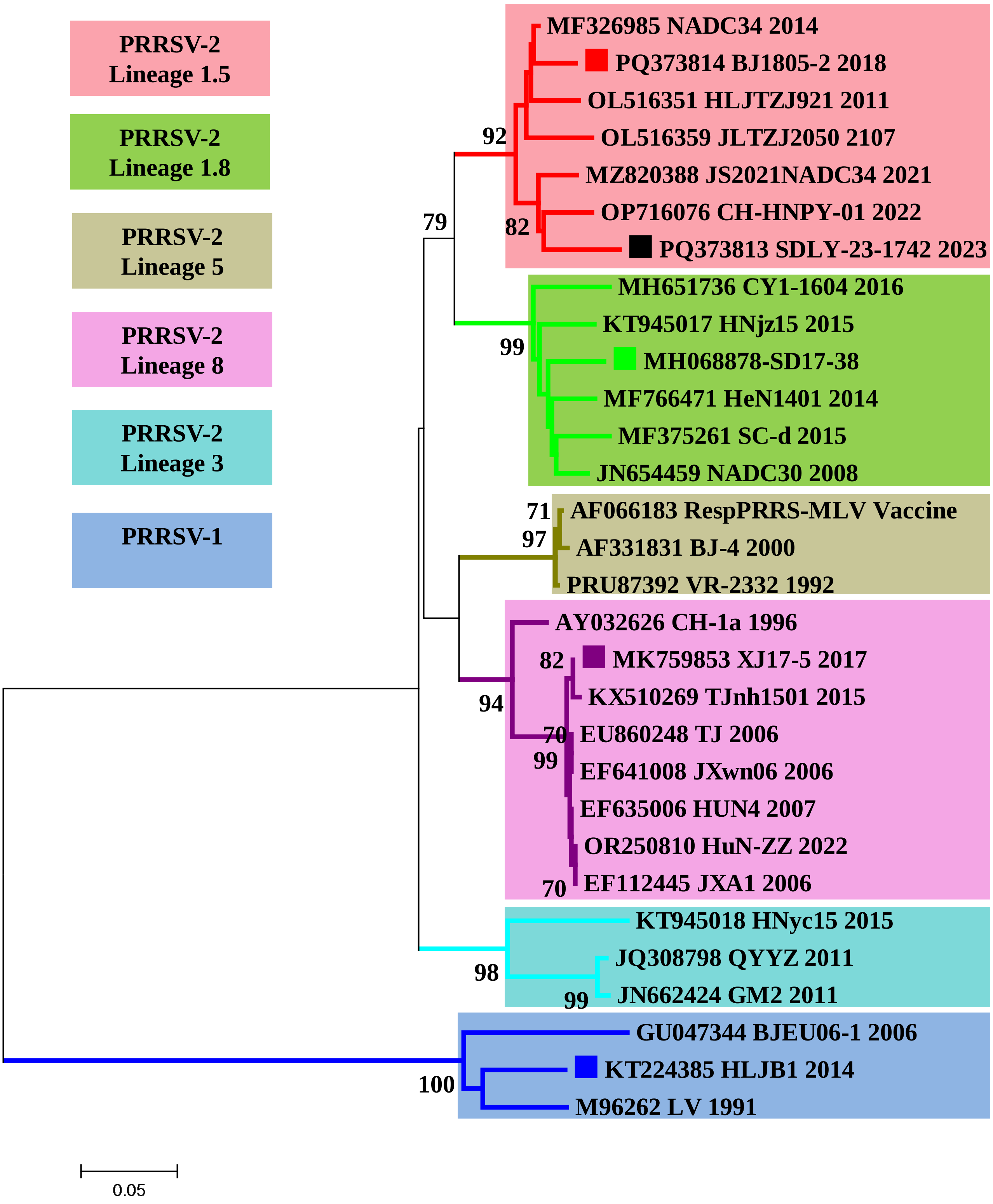

FIG S4 ORF5-based phylogenetic tree was constructed using 30 representative PRRSV strains. Distinct PRRSV species and lineages are highlighted in different colors. The five strains used in this study are remarked in color squares. Each virus is presented by the GenBank accession number, virus name, and year of isolation. Bootstrap values from 1000 replicates are indicated for each node.

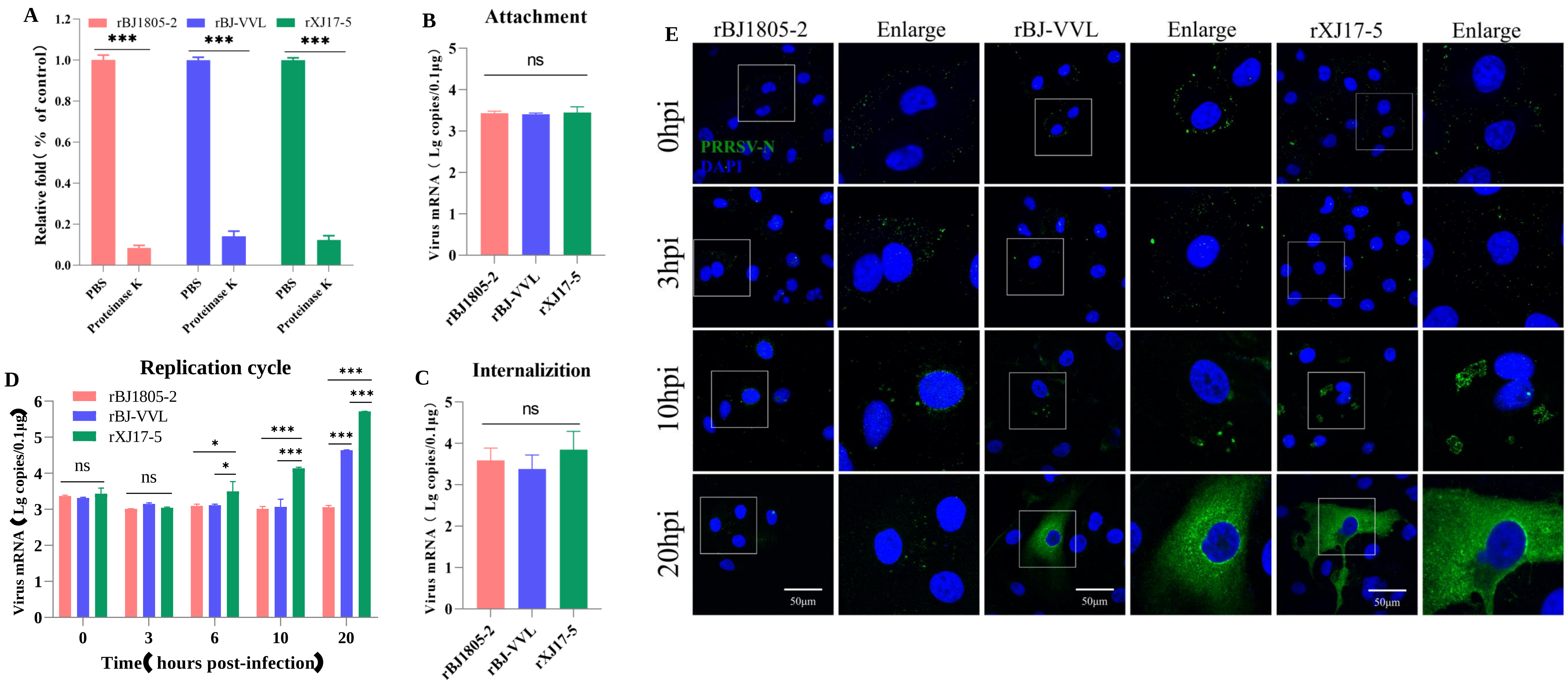

FIG S5 The influences of GP2a 91/97/98 aa substitutions on PRRSV replication cycle. (A) The effects of Proteinase K treatment to eliminate attached but not internalized PRRSV particles. (B) The influence of GP2a 91/97/98 aa substitutions on PRRSV attachment. (C) The influence of GP2a 91/97/98 aa substitutions on PRRSV internalization. (D) The influence of GP2a 91/97/98 aa substitutions on PRRSV replication at 0, 3, 6, 10, 20 hpi, respectively. (E) Representative IFA results for the influences of GP2a 91/97/98 aa substitutions on PRRSV replication at 0, 3, 10, 20 hpi, respectively.

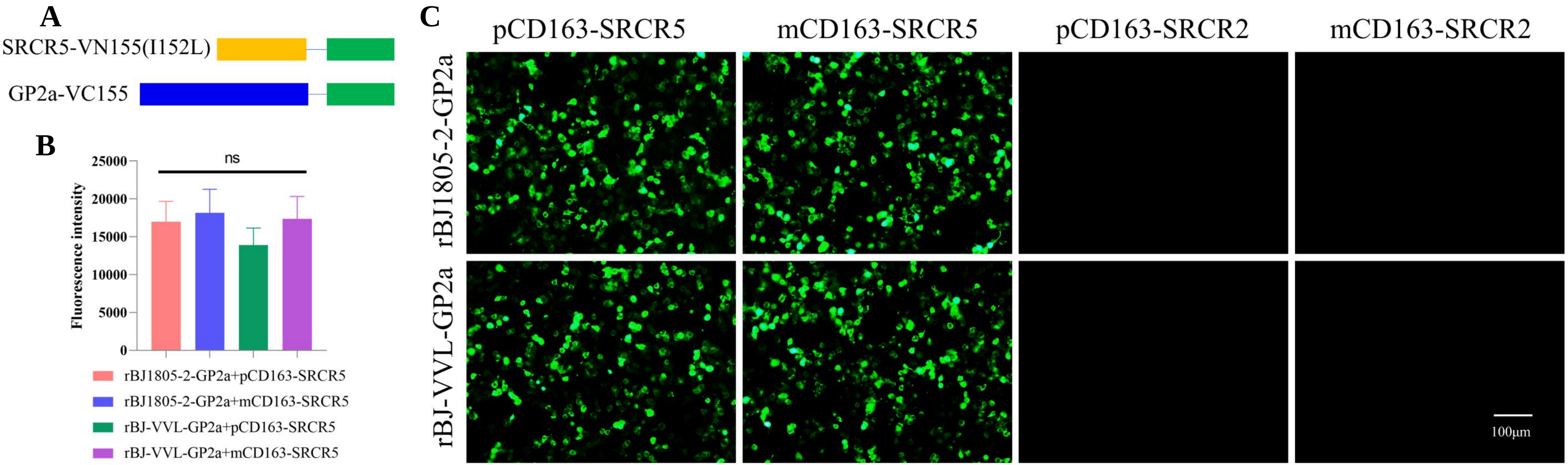

FIG S6 The influences of GP2a 91/97/98 aa substitutions on the interactions between PRRSV-GP2a and CD163-SRCR5. (A) The scheme diagram for the BiFC assay constructs between PRRSV GP2a and CD163 SRCR5. (B) Relative fluorescence intensities resulted from BiFC screening for GP2a (from rBJ1805-2 and rBJ-VVL) and SRCR5 (from porcine CD163 and monkey CD163) interactions. (C) BiFC screening images for GP2a (from rBJ1805-2 and rBJ-VVL) and SRCR5 (from porcine CD163 and monkey CD163) interactions. The interactions between GP2a with SRCR2 were set as negative control.

Table S1. Primers used for the constructions of PRRSV infectious clones

Primer name	Sequence(5'-3')
Primer of pACYC177-rBJ1805-2 infectious clone	
rBJ1805-2-PacI-F1	AGCTCGttaattaaTACATGAAGTATAGGTGTTGGCTCT
rBJ1805-2-Bsu36I-R1	AATAATACTAAAGCTAAGATTCCTGAGGGCATGTTCACGGAAATCAAGAA
rBJ1805-2-Bsu36I-F2	TTCTTGATTTCCGTGAACATGCCCTCAGGAATCTTAGCTTTAGTATTATT
rBJ1805-2-XbaI-R2	ACCATTCAGGGTCGTACTCTAGACAAGCACAACTATCCAT
rBJ1805-2-XbaI-F3	ATGGATAGTTGTGCTTGTCTAGAGTACGACCCTGAATGGT
rBJ1805-2-1R3	AGCGAGGAGGCTGGGACCATGCCGGCCtttttttttttttttttttttAATTTCGGCCGCATGGTTCT
rBJ1805-2-AscI-2R3	ACAGGGCGCGCCGTCCCATTCGCCATTACCGAGGGGACGGTCCCCTCGGAATGTT<br>GCCCAGCCGGCGCCAGCGAGGAGGCTGGGACCAT
Primers of pACYC177-rSD17-38 infectious clone	
SD17-38-PacI-F1	TATATAAGCAGAGCTCGTTAATTAATACATGACGTATAGGTGTTGGCTCCA
SD17-38-ScaI-R1	TTGCCATCGGCGGGTGGGGAGTAGTACTTAAGGGGTTCATCCTT
SD17-38-ScaI-F2	AAGGATGAACCCCTTAAGTACTACTCCCCACCCGCCGATGGCAA
SD17-38-NotI-R2	TGGCATTAGGCGTAAGGCAGGCGGCCGCATGTATTGGGCTCCCAGTAT
SD17-38-NotI-F3	ATACTGGGAGCCCAATACATGCGGCCGCCTGCCTTACGCCTAATGCCA
SD17-38-Bsp1407I-R3	AACAGAGGAAAATATGGAGGAATGTACAGCTATTAACCATTGCTGAAAGT
SD17-38-Bsp1407I-F4	ACTTTCAGCAATGGTTAATAGCTGTACATTCCTCCATATTTTCCTCTGTT
SD17-38-4R1	AATGTTGCCCAGCCGGCGCCAGCGAGGAGGCTGGGACCATGCCGGCCTTTTTTTTTT<br>TTTTTTTTTTTAATTTCGGCCGCATGGTTCT
SD17-38-AscI-4R2	TCGCTGGCGCCGGCTGGGCAACATTCCGAGGGGACCGTCCCCTCGGTAATGGCGA<br>ATGGGACGGCGCGCCCTGTGCCTTCTAGTTGCCA
Primers of pACYC177-rBJ1805-2 chimeric clone	
rBJ-XbaI-F3	CGCTGCAATACTCATGGATAGTTGTGCTTGT
rBTX234-ORF2-F	CAACGTTGGGCCTGGACTGAaatgaaatggggtctatgcaaagcc
rBTX234-ORF1b-R	ggctttgcatagaccccatttcattTCAGTCCAGGCCCAACGTTG
rBTX234-ORF5-F	gccatcctactggcaatttgaATGTTCAGGTATGTTGG
rBTX234-ORF4-R	CCAACATACCTGAACATtcaaattgccagtaggatggc
rBTX23-ORF3-F	ctcagtgccgcacggcgatagGAACACCCGTTTACATCA
rBTX23-ORF3-R	TGATGTAAACGGGTGTTCctatcgccgtgcggcactgag
rBTX4-ORF4-F	ggcaattggtttcacctggaATGGCTGCGTCCTTTC
rBTX4-ORF4-R	GAAAGGACGCAGCCATtccaggtgaaaccaattgcc
rBTX2-ORF3-F	gaactcatggtgaATTACACGGTGTGCCAG
rBTX2-ORF3-R	CTGGCACACCGTGTAATtcaccatgagttc
rBTX3-ORF3-F	CAGCAatggctaatagctgtacattcctcc
rBTX3-ORF3-R	ggaggaatgtacagctattagccatTGCTG
rBTX4-ORF4-F	GCAATTGGTTTCACCTAGAatggctacg
rBTX4-ORF4-R	cgtagccatTCTAGGTGAAACCAATTGC
rBTX2-1-191aa-F	cagcaATGGCTAATAGCTGTACACTCCTC
rBTX2-1-191aa-R	GAGGAGTGTACAGCTATTAGCCATtgctg
rBTX2-1-76aa-F	caccctgagcaattacagaagatcttatgaGGTCTTC
rBTX2-1-76aa-R	GAAGACCtcataagatcttctgtaattgctcagggtg

rBTX2-77-191aa-F	CTGAGCAGTTACAGAAGATCCTATGAggcctttc
rBTX2-77-191aa-R	gaaaggccTCATAGGATCTTCTGTAACTGCTCAG
rBTX2-77-98-118-191aa-F	GTCAACCCTGATTGATGAAATGGTGTCGC
rBTX2-77-98-118-191aa-R	GCGACACCATTTCATCAATCAGGGTTGAC
rBTX2-77-88aa-F	gtgtcaggtagaTatccccaccTGGGGAACCAAGCAC
rBTX2-77-88aa-R	GTGCTTGGTTCCCCAggtggggatAtctacctgacac
rBJ-VVL-F	CGTCTGGGGAgtCAAGCACCCCCTGGGAgTGcTTTGGCACCACAAG
rBJ-VVL-R	CTTGTGGTGCCAAAgCAcTCCCAGGGGGTGCTTGacTCCCCAGACG
rBJ-VVL-V91T-F	CGTCTGGGGAACCAAGCACCCCCTGGGAgTGcTTTGGCACCACAAG
rBJ-VVL-V91T-R	CTTGTGGTGCCAAAgCAcTCCCAGGGGGTGCTTGGTTCCCCAGACG
rBJ-VVL-V97M-F	CGTCTGGGGAgtCAAGCACCCCCTGGGAATGcTTTGGCACCACAAG
rBJ-VVL-V97M-R	CTTGTGGTGCCAAAgCATTCCCAGGGGGTGCTTGacTCCCCAGACG
rBJ-VVL-L97F-F	CGTCTGGGGAgtCAAGCACCCCCTGGGAgTGTTTTGGCACCACAAG
rBJ-VVL-L97R-F	CTTGTGGTGCCAAAACAcTCCCAGGGGGTGCTTGacTCCCCAGACG
rBTA-FIL-F	CGTCTGGGGAttCAAGCACCCCCTGGGAaTacTTTGGCACCACAAG
rBTA-FIL-R	CTTGTGGTGCCAAAgtAtTCCCAGGGGGTGCTTGaaTCCCCAGACG
rBJ-VVL-reverse-F	CGTCTGGGGAACCAAGCACCCCCTGGGAATGTTTTGGCACCACAAG
rBJ-VVL-reverse-R	CTTGTGGTGCCAAAACATTCCCAGGGGGTGCTTGGTTCCCCAGACG
Primers of pACYC177-rSD17-38 chimeric clone	
rSD17-38-F3	cctagtgatactgggagcccaatacatgc
rSTX234-ORF2-F	ggcttgaactgaaatgaaatggggtctatgc
rSTX234-ORF1b-R	gcatagaccccatttcatttcagttcaagcc
rSTX234-ORF5-F	gccatcctactggcaatttgaATGTTTAGATATGTTGG
rSTX234-ORF4-R	CCAACATATCTAAACATtcaaattgccagtaggatggc
rSD-VVL-F	ccttcctgggggGTaaaacatcccttgggtGtgCtttggcaccataag
rSD-VVL-R	cttatggtgccaaaGcaCacccaagggatgttttACcccccaggaagg
rSD-TVL-F	ccttcctgggggACaaaacatcccttgggtGtgCtttggcaccataag
rSD-TVL-R	cttatggtgccaaaGcaCacccaagggatgttttGTcccccaggaagg
rSD-VML-F	ccttcctgggggGTaaaacatcccttgggtAtgCtttggcaccataag
rSD-VML-R	cttatggtgccaaaGcaTacccaagggatgttttACcccccaggaagg
rSD-VVF-F	ccttcctgggggGTaaaacatcccttgggtGtgTtttggcaccataag
rSD-VVF-R	cttatggtgccaaaAcaCacccaagggatgttttACcccccaggaagg
rSTA-FIL-F	ccttcctgggggTTCaaacatcccttgggtAtACtttggcaccataag
rSTA-FIL-R	cttatggtgccaaaGTaTacccaagggatgtttGAAcccccaggaagg
Primers of pACYC177-rXJ17-5 chimeric clone	
rXJ17-5-AscI-F	ggaggattacaatgatgcgtttcggg
rXJ17-5-AscI-F3-F1	GTTTCGGGCGCGCCAGAAAGGGAAAATTTATAAAGCTAATGCCACCAG CATGAGGTTTCATTTTCCCCCGGGCCCTGTCATTGAACCAA
rXTB234-ORF2-F	cgggccctgtcattgaaccaactttaggcctgaattgaaATGAAATGGGTGCT
rXTB234-ORF4-F	GCCATCCTGCTGGCGATTTGAatgttcaagtatgttgg
rXTB234-ORF4-R	ccaacatacttgaacatTCAAATCGCCAGCAGGATGGC
rXTB-nsp2-F	ggcagtcataagtggtacggtGCTGCAAAGAGAAC
rXTB-nsp1-R	GTTCTCTTTGCAGCaccgtaccacttatgactgcc

rXTB-nsp3-F	CAAGTCCTCTGGGGGAGGCccacacctcatt
rXTB-nsp2-R	aatgaggtgtggGCCTCCCCCAGAGGACTTG
XJ17-5-AflⅡ-fusion-R	atcatccggggcaggaaggcataggtgcttaag
rXTB-M34-a-F	CCTTGTGAattacacggtatgcccgctttg
rXTB-M34-a-R	caaagcgggcataccgtgtaatTCACAAGG
rXJ-TMF-F	ccacctggggcACcaaacacccctttggggAtgTtttggcaccataagg
rXJ-TMF-R	ccttatggtgccaaaAcaTccccaaagggtgtttgGTgccccaggtgg
rXTB-M34-b-F	gaactcatggtgaattacacggtGtgc
rXTB-M34-b-R	gcaCaccgtgtaattcaccatgagttc
rXTB-M23-TMF-d-F	ggcaattggtttcacctggaATGGCTGCGTCCTTTC
rXTB-M23-TMF-d-R	GAAAGGACGCAGCCATtccaggtgaaaccaattgcc
rXTB-M34-c-F	GTGAATTACACGGTatgcccgctttgcc
rXTB-M34-c-R	ggcaaagcgggcatACCGTGTAATTCAC
rXTB-TMF-56-ORF5-F	gccatcctactggcaATTTGAATGTTCAGG
rXTB-TMF-56-ORF4-R	CCTGAACATTCAAATtGCCAGtAGGATGGC
rXTB-TMF-56-ORF7-F	atgccaaataacaacggcaagcagc
rXTB-TMF-56-ORF6-R	gctgcttgccgttgttatttggcat
Primers of pACYC177-rHLJB1 chimeric clone	
HLJB1-BglII-F3	agtgtcagtcaccggttggggctggtag
rHLJB1-FIL-F	caatttgcaTtcaagcatccattaggtatACtttggcacatgcgag
rHLJB1-FIL-R	ctcgcatgtgccaaaGTatacctaatggatgcttgaAtgcaaattg
rHTA23-ORF2-F	ATGCAATGGGGTTACTGTGGAGTAAAATTAGCCAGCTGTT
rHTA23-ORF1b-R	AACAGCTGGCTAATTTTACTCCACAGTAACCCCATTGCAT
rHTA23-ORF3-F	CACATTAGCCGTTATCccgatgaatcgtatttgtacaacg
rHTA23-ORF3-R	cgttgtacaaatacgattcatcggTCACATTAGCCGTTATC
rHTA2-F	GCAACACATCATTCGAGCTAACCATCAACT
rHTA2-R	AGTTGATGGTTAGCTCGAATGATGTGTTGC
rHTA3-F	gaccgacttcagacaATGGCTCATCAGTGT
rHTA3-R	ACACTGATGAGCCATtgtctgaagtcggtc
rHTA-FIL-3-1-181aa-F	caattggttccatttggaATGGCTGCGGCCATTC
rHTA-FIL-3-1-181aa-R	GAATGGCCGCAGCCATtccaaatggaaccaattg
Primers in pACYC177 vector	
pACYC177-PAC1-fusion-F	GTGTACGGTGGGAGGTCTATATAAGCAGAGCTCG
rBJ-NOT1-2fu-1	CAAACAACAGATGGCTGGCAACTAGAAGGCACAG

Table S2. The construction strategies for chimeric viruses.

NO.	Chimeric virus	1st primer pairs	Amplicon 1	2nd primer pairs	Amplicon 2	Chimeric plasmid
1	rBTX2	rBJ-XbaI-F + rBTX2-ORF3-R	pACYC177-rBTX234	rBTX2-ORF3-F + rBJ-NOT1-2fu-1	pACYC177-rBJ1805-2	pACYC177-rBTX2
2	rBTX23	rBJ-XbaI-F + rBTX23-ORF3-R	pACYC177-rBTX234	rBTX23-ORF3-F + rBJ-NOT1-2fu-1	pACYC177-rBJ1805-2	pACYC177-rBTX23
3	rBTX34	rBJ-XbaI-F + rBTX3-ORF3-R	pACYC177-rBJ1805-2	rBTX3-ORF3-F + rBJ-NOT1-2fu-1	pACYC177-rBTX234	pACYC177-rBTX34
4	rBTX4	rBJ-XbaI-F + rBTX4-ORF4-R	pACYC177-rBJ1805-2	rBTX4-ORF4-F + rBJ-NOT1-2fu-1	pACYC177-rBTX234	pACYC177-rBTX4
5	rBTX3	rBJ-XbaI-F + rBTX3-ORF3-R	pACYC177-rBJ1805-2	rBTX3-ORF3-F + rBJ-NOT1-2fu-1	pACYC177-rBTX23	pACYC177-rBTX3
6	rBTX24	rBJ-XbaI-F + rBTX4-ORF4-R	pACYC177-rBTX2	rBTX4-ORF4-F + rBJ-NOT1-2fu-1	pACYC177-rBTX4	pACYC177-rBTX24
7	rBTX2-1-191aa	rBJ-XbaI-F + rBTX2-1-191aa-R	pACYC177-rBTX2	rBTX2-1-191aa-F + rBJ-NOT1-2fu-1	pACYC177-rBJ1805-2	pACYC177-rBTX2-1-191aa
8	rBTX2-77-191aa	rBJ-XbaI-F + rBTX2-77-191aa-R	pACYC177-rBTX2-1-191aa	rBTX2-77-191aa-F + rBJ-NOT1-2fu-1	pACYC177-rBJ1805-2	pACYC177-rBTX2-77-191aa
9	rBTX2-1-76aa	rBJ-XbaI-F + rBTX2-1-76aa-R	pACYC177-rBTX2-1-191aa	rBTX2-1-76aa-F + rBJ-NOT1-2fu-1	pACYC177-rBJ1805-2	pACYC177-rBTX2-1-76aa
10	rBTX2-1-76 + 202-256aa	rBJ-XbaI-F + rBTX3-ORF3-R	pACYC177-rBTX2-1-76aa	rBTX3-ORF3-F + rBJ-NOT1-2fu-1	pACYC177-rBTX3	pACYC177-rBTX2-1-76 + 202-256aa
11	rBTX2-77-98aa	rBJ-XbaI-F + rBTX2-77-98-118-191aa-R	pACYC177-rBTX2-77-191aa	rBTX2-77-98-118-191aa-F + rBJ-NOT1-2fu-1	pACYC177-rBJ1805-2	pACYC177-rBTX2-77-98aa
12	rBTX2-118-191aa	rBJ-XbaI-F + rBTX2-77-98-118-191aa-R	pACYC177-rBJ1805-2	rBTX2-77-98-118-191aa-F + rBJ-NOT1-2fu-1	pACYC177-rBTX2-77-191aa	pACYC177-rBTX2-118-191aa
13	rBTX2-77-88aa	rBJ-XbaI-F + rBTX2-77-88aa-R	pACYC177-rBTX2-77-191aa	rBTX2-77-88aa-F + rBJ-NOT1-2fu-1	pACYC177-rBJ1805-2	pACYC177-rBTX2-77-88aa
14	rBTX2-77-88 + 118-191aa	rBJ-XbaI-F + rBTX2-77-98-118-191aa-R	pACYC177-rBTX2-77-88aa	rBTX2-77-98-118-191aa-F + rBJ-NOT1-2fu-1	pACYC177-rBTX2-118-191aa	pACYC177-rBTX2-77-88 + 118-191aa
15	rBJ-VVL	rBJ-XbaI-F + rBJ-VVL-R	pACYC177-rBJ1805-2	rBJ-VVL-F + rBJ-NOT1-2fu-1	pACYC177-rBJ1805-2	pACYC177-rBJ-VVL
16	rBJ-TVL	rBJ-XbaI-F + rBJ-TVL-R	pACYC177-rBJ1805-2	rBJ-TVL-F + rBJ-NOT1-2fu-1	pACYC177-rBJ1805-2	pACYC177-rBJ-TVL
17	rBJ-VML	rBJ-XbaI-F + rBJ-VML-R	pACYC177-rBJ1805-2	rBJ-VML-F + rBJ-NOT1-2fu-1	pACYC177-rBJ1805-2	pACYC177-rBJ-VML
18	rBJ-VVF	rBJ-XbaI-F + rBJ-VVF-R	pACYC177-rBJ1805-2	rBJ-VVF-F + rBJ-NOT1-2fu-1	pACYC177-rBJ1805-2	pACYC177-rBJ-VVF
19	rBTX2-TMF	rBJ-XbaI-F + rXJ-TMF-R	pACYC177-rBTX2	rXJ-TMF-F + rBJ-NOT1-2fu-1	pACYC177-rBTX2	pACYC177-rBTX2-TMF
20	rBTX23-TMF	rBJ-XbaI-F + rXJ-TMF-R	pACYC177-rBTX23	rXJ-TMF-F + rBJ-NOT1-2fu-1	pACYC177-rBTX23	pACYC177-rBTX23-TMF
21	rBTX24-TMF	rBJ-XbaI-F + rXJ-TMF-R	pACYC177-rBTX24	rXJ-TMF-F + rBJ-NOT1-2fu-1	pACYC177-rBTX24	pACYC177-rBTX24-TMF
22	rBJ-FIL	rBJ-XbaI-F + rBJ-FIL-R	pACYC177-rBJ1805-2	rBJ-FIL-F + rBJ-NOT1-2fu-1	pACYC177-rBJ1805-2	pACYC177-rBJ-FIL
23	rBJ-VVL-Reverse	rBJ-XbaI-F + rBJ-VVL-TMF-R	pACYC177-rBJ-VVL	rBJ-TMF-F + rBJ-NOT1-2fu-1	pACYC177-rBJ-VVL	pACYC177-rBJ-VVL-Reverse
24	rBJ-FIL-Reverse	rBJ-XbaI-F + rBJ-VVL-TMF-R	pACYC177-rBJ-FIL	rBJ-TMF-F + rBJ-NOT1-2fu-1	pACYC177-rBJ-FIL	pACYC177-rBJ-FIL-Reverse
26	rSD-VVL	rSD17-38-F3 + rSD-VVL-R	pACYC177-rSD17-38	rSD-VVL-F + rBJ-NOT1-2fu-1	pACYC177-rSD17-38	pACYC177-rSD-VVL
27	rSTX234-TMF	rSD17-38-F3 + rXJ-TMF-R	pACYC177-rSTX234	rXJ-TMF-F + rBJ-NOT1-2fu-1	pACYC177-rSTX234	pACYC177-rSTX234-TMF
28	rSD-TVL	rSD17-38-F3 + rSD-TVL-R	pACYC177-rSD17-38	rSD-TVL-F + rBJ-NOT1-2fu-1	pACYC177-rSD17-38	pACYC177-rSD-TVL
29	rSD-VML	rSD17-38-F3 + rSD-VVL-R	pACYC177-rSD17-38	rSD-VML-F + rBJ-NOT1-2fu-1	pACYC177-rSD17-38	pACYC177-rSD-VML
30	rSD-VVF	rSD17-38-F3 + rSD-VVL-R	pACYC177-rSD17-38	rSD-VVF-F + rBJ-NOT1-2fu-1	pACYC177-rSD17-38	pACYC177-rSD-VVF
31	rSD-FIL	rSD17-38-F3 + rSD-VVL-R	pACYC177-rSD17-38	rSD-VVL-F + rBJ-NOT1-2fu-1	pACYC177-rSD17-38	pACYC177-rSD-FIL
32	rXTB234-VVL	rXJ17-5-AscI-F + rBJ-VVL-R	pACYC177-rXTB234	rBJ-VVL-F + rBJ-NOT1-2fu-1	pACYC177-rXTB234	pACYC177-rXTB234-VVL
33	rXJ-TMF	rXJ17-5-AscI-F + rXJ-TMF-R	pACYC177-rXJ17-5	rXJ-TMF-R + rBJ-NOT1-2fu-1	pACYC177-rXJ17-5	pACYC177-rXJ-TMF
34	rXTB-M2-TMF	rXJ17-5-AscI-F + rBTX2-ORF3-R	pACYC177-rXJ-TMF	rBTX2-ORF3-F + rBJ-NOT1-2fu-1	pACYC177-rXTB234	pACYC177-rXTB-M2-TMF
35	rXTB-M3	rXJ17-5-AscI-F + rBTX3-ORF3-R	pACYC177-rXTB234	rBTX3-ORF3-F + rBJ-NOT1-2fu-1	pACYC177-rXTB-M23-TMF	pACYC177-rXTB-M3
36	rXTB-M4	rXJ17-5-AscI-F + rBTX4-ORF4-R	pACYC177-rXTB234	rBTX4-ORF4-F + rBJ-NOT1-2fu-1	p pACYC177-rXJ-TMF	pACYC177-rXTB-M4

37	rXTB-M23-TMF	rXJ17-5-AscI-F + rBTX23-ORF3-R	pACYC177-rXJ-TMF	rBTX23-ORF3-F + rBJ-NOT1-2fu-1	pACYC177-rXTB234	pACYC177-rXTB-M23-TMF
38	rXTB-M24-TMF	rXJ17-5-AscI-F + rBTX4-ORF4-R	pACYC177-rXTB-M2-TMF	rBTX4-ORF4-F + rBJ-NOT1-2fu-1	pACYC177-rXTB-M4	pACYC177-rXTB-M24-TMF
39	rXTB-M34	rXJ17-5-AscI-F + rBTX3-ORF3-R	pACYC177-rXTB234	rBTX3-ORF3-F + rBJ-NOT1-2fu-1	pACYC177-rXJ-TMF	pACYC177-rXTB-M34
40	rXTB-M23-TMF-a	rXJ17-5-AscI-F + rBTX2-77-191aa-R	pACYC177-rXTB234	rBTX2-77-191aa-F + rBJ-NOT1-2fu-1	pACYC177-rXTB-M2-TMF-1-191aa	pACYC177-rXTB-M23-TMF-a
41	rXTB-M23-TMF-b	rXJ17-5-AscI-F + rBTX2-1-76aa-R	pACYC177-rXJ-TMF	rBTX2-1-76aa-F + rBJ-NOT1-2fu-1	pACYC177-rXTB-M3	pACYC177-rXTB-M23-TMF-b
42	rXTB-M2-TMF-1-191aa	rXJ17-5-AscI-F + rBTX2-1-191aa-R	pACYC177-rXJ-TMF	rBTX2-1-191aa-F + rBJ-NOT1-2fu-1	pACYC177-rXTB234	pACYC177-rXTB-M2-TMF-1-191aa
43	rXTB-M23-TMF-c	rXJ17-5-AscI-F + rBTX4-ORF4-R	pACYC177-rXTB-M2-TMF-1-191aa	rBTX4-ORF4-F + rBJ-NOT1-2fu-1	pACYC177-rXTB-M3	pACYC177-rXTB-M23-TMF-c
44	rXTB-M23-TMF-d	rXJ17-5-AscI-F + rXTB-M23-TMF-d-R	pACYC177-rXTB-M23-TMF	rXTB-M23-TMF-d-F + rBJ-NOT1-2fu-1	pACYC177-rXTB234	pACYC177-rXTB-M23-TMF-d
45	rXTB-M34-a	rXJ17-5-AscI-F + rXTB-M34-a-R	pACYC177-rXTB234	rXTB-M34-a-F + rBJ-NOT1-2fu-1	pACYC177-rXTB-M34	pACYC177-rXTB-M34-a
46	rXTB-M34-b	rXJ17-5-AscI-F + rXTB-M34-b-R	pACYC177-rXTB-M3	rXTB-M34-b-F + rBJ-NOT1-2fu-1	pACYC177-rXTB-M4	pACYC177-rXTB-M34-b
47	rXTB-M3-1-181aa	rXJ17-5-AscI-F + rBTX3-ORF3-R	pACYC177-rXTB234	rBTX3-ORF3-F + rBJ-NOT1-2fu-1	pACYC177-rXTB-M23-TMF-d	pACYC177-rXTB-M3-1-181aa
48	rXTB-M34-c	rXJ17-5-AscI-F + rXTB-M34-c-R	pACYC177-rXTB-M3-1-181aa	rXTB-M34-c-F + rBJ-NOT1-2fu-1	pACYC177-rXJ-TMF	pACYC177-rXTB-M34-c
49	rHLJB1-FIL	rHLJB1-BglII-F3 + rHLJB1-FIL-R	pACYC177-rHLJB1	rHLJB1-FIL-F + rBJ-NOT1-2fu-1	pACYC177-rHLJB1	pACYC177-rHLJB1-FIL
50	rHTA2	rHLJB1-BglII-F3 + rHTA2-R	pACYC177-rHTA23	rHTA2-F + rBJ-NOT1-2fu-1	pACYC177-rHLJB1	pACYC177-rHTA2
51	rHTA3	rHLJB1-BglII-F3 + rHTA3-R	pACYC177-rHLJB1	rHTA3-F + rBJ-NOT1-2fu-1	pACYC177-rHTA23	pACYC177-rHTA3
52	rHTA-FIL-3	rHLJB1-BglII-F3 + rHTA3-R	pACYC177-rHLJB1-FIL	rHTA3-F + rBJ-NOT1-2fu-1	pACYC177-rHTA23	pACYC177-rHTA-FIL-3
53	rHTA-FIL-3-1-181aa	rHLJB1-BglII-F3 + rHTA-FIL-3-182-270aa-R	pACYC177-rHTA-FIL-3	rHTA-FIL-3-182-270aa-F + rBJ-NOT1-2fu-1	pACYC177-rHLJB1	pACYC177-rHTA-FIL-3-1-181aa
54	rHTA-FIL-3-182-270aa	rHLJB1-BglII-F3 + rHTA-FIL-3-182-270aa-R	pACYC177-rHLJB1-FIL	rHTA-FIL-3-182-270aa-F + rBJ-NOT1-2fu-1	pACYC177-rHTA3	pACYC177-rHTA-FIL-3-182-270aa
55	rHLJB1-VVL	rHLJB1-BglII-F3 + rHLJB1-VVL-R	pACYC177-rHLJB1	rHLJB1-VVL-RF + rBJ-NOT1-2fu-1	pACYC177-rHLJB1	pACYC177-rHLJB1-VVL

* The 1st primer pairs were used for amplicon 1 amplification. The 2nd primer pairs were used for amplicon 2 amplification. The corresponding amplicons 1 and 2 were seamlessly cloned into vectors to obtain chimeric plasmids. The chimeric plasmids were transferred into BHK-21 cells to generate corresponding chimeric viruses.
